# Knowns and unknowns of plastic waste flows in the Netherlands

**DOI:** 10.1177/0734242X231180863

**Published:** 2023-07-16

**Authors:** Delphine Lobelle, Li Shen, Bas van Huet, Tim van Emmerik, Mikael Kaandorp, Giulia Iattoni, Cornelius Peter Baldé, Kara Lavender Law, Erik van Sebille

**Affiliations:** 1 Institute for Marine and Atmospheric Research, Utrecht University, Utrecht, The Netherlands; 2 Fugro, Leidschendam, The Netherlands; 3 Copernicus Institute of Sustainable Development, Utrecht University, Utrecht, The Netherlands; 4 Rijkswaterstaat, Utrecht, The Netherlands; 5 Hydrology and Quantitative Water Management Group, Wageningen University, Wageningen, The Netherlands; 6 United Nations University, Vice Rectorate in Europe, Sustainable Cycles Programme (SCYCLE), Bonn, Germany; 7 Sea Education Association, Falmouth, MA, USA; 8 Centre for Complex Systems Studies, Utrecht University, Utrecht, The Netherlands; 9 Freudenthal Institute, Utrecht University, Utrecht, The Netherlands

**Keywords:** Material flow analysis, plastic waste, Netherlands, waste management, mismanagement, plastic pollution

## Abstract

Plastic entering the environment is a growing threat for ecosystems. We estimate the annual mass of known Dutch plastic waste generated and littered and where it ends up. We use two methods: (1) a material flow analysis of plastic waste separately collected from 13 economic sectors (including households, industry and imports) and estimate the amount sent to processing plants or exported and (2) a mismanagement model from observations of litter (on Dutch beaches and riverbanks) plus estimates of inadequately managed exported plastic scraps entering the environment abroad. In 2017 (the most recent complete data set available), an estimate of 1990 (±111) kilotonnes [kt] of plastic waste was separately collected. The top three plastic waste generating sectors (74% of the total) were households, clothing and textiles, and importation. Our mismanagement model estimates that 4.3–21.2 kt enters the environment annually; almost all of which occurs in foreign countries after inadequate management of imported Dutch waste. We highlight unknowns, including the source and/or destination of imported (623 kt) and exported (514 kt) plastics, plastics in non-household mixed waste streams and the plastic fraction of some separately collected waste, for example, e-waste. Our results stress the need for improved monitoring and reporting of plastic waste. Beyond the Netherlands, our recommendations could also help other high-income countries’ decision-makers reach their circular economy goals.

## Introduction

Plastics ending up in the environment are a global problem that threaten ecosystem health. It has been shown that countries of all economic levels of development are responsible for plastic pollution (Geyer et al., 2017; Jambeck et al., 2015). Although plastics are extremely useful, light, durable, strong and flexible materials, the poor management of end-of-life plastics is concerning and inadmissible. With ‘business-as-usual’ emissions of plastics into aquatic ecosystems predicted to double in the next 10 years (Borrelle et al., 2020), new targets have been set in recent years to curb this fate. For example, the European Green Deal’s Strategy for Plastics in the Circular Economy (European Commission, 2018) states that by 2030, all European Union (EU) plastic packaging placed on the market must either be reusable or be recycled cost-effectively. The European Commission’s ‘Mission Starfish 2030’ to Restore our Ocean and Waters is also working towards a zero marine pollution target (Lamy et al., 2020).

The Netherlands has an ambitious circular plastic goal. The Plastic Pact NL (signed by 75 parties in February 2019) declares that one of the main targets is to ensure that new single-use products are 100% recyclable, 70% is recycled and they have at least 35% of recycled content by 2025 (Plastic Pact NL, 2021; van Veldhoven-van der Meer, 2019). The Dutch government is also striving for circularity of plastic production, use and disposal in the Netherlands by 2050 (Dutch Government, 2016). To reach such goals, Plastic Pact members and the authorities require data to monitor the mass flow of plastic waste, especially how much is properly managed and how much ends up in the environment.

Recent studies have assessed plastic waste flows on national and global scales (Brouwer et al., 2019; González-Fernández et al., 2021; Jambeck et al., 2015; Kawecki et al., 2018; Meijer et al., 2021; Snijder and Nusselder, 2019). Brouwer et al. (2018, 2019) present a material flow analysis (MFA) of specifically post-consumer Dutch plastic packaging waste. CE Delft (Snijder and Nusselder, 2019) and The Netherlands Organisation for applied scientific research (Wijngaard et al., 2020) produced reports for an MFA with numerous categories of Dutch plastic waste. Since these studies were mainly based on secondary data, here we aim to generate an overview based largely on primary sources (e.g. data from the Directorate-General for Public Works and Water Management, Rijkswaterstaat (RWS)). Our analysis, based on high-quality data with quantified uncertainties, also includes more source categories of waste, imports and exports of plastic waste and an estimate of mismanagement (plastic that enters the environment).

A few methods have been developed in recent years for plastic waste entering the environment. The model by Jambeck et al. (2015) uses World Bank statistics of each country’s solid waste generation and management, coastal population density and economic status. The Netherlands ranked 20th place globally in their plastic waste generation estimates (1385 kt in 2010, where 1 kt = 1,000,000 kg). Their estimated littering amount was 27.7 kt (2%; which was the assumed minimum littering rate for every country). Other models (Lebreton et al., 2017; Meijer et al., 2021; Schmidt et al., 2017) estimating global plastic emissions from rivers to oceans use different assumptions on geographical contribution and use available field data to tune and validate their models. Meijer et al. (2021) also include a probabilistic analysis, estimating that 0.27 kt year^−1^ enters the North Sea from Dutch rivers annually. Other studies have also considered plastic entering the environment after inadequate management of imported plastic waste originating from the United States (Law et al., 2020) and Europe (Bishop et al., 2020). The novelty we present is to base our analysis on observational data from Dutch plastic monitoring projects (e.g. The North Sea Foundation beach and riverbank plastic monitoring), in combination with plastic waste ending up in the environment after inadequate management in importing countries, to estimate the known Dutch plastic mismanagement.

The objective of our study is to provide an overview of the knowns and unknowns of managed and mismanaged plastic waste flows in the Netherlands in 2017, the most recent year for which most data are available. We combine two complementary approaches: (1) an MFA to estimate the plastic waste generation and destination from the most essential economic sectors in the Netherlands (e.g. the amount of collected post-industrial waste that is sent to be recycled) and (2) a mismanagement model by extrapolating in situ plastic litter data from beaches and rivers to the national scale, as well as foreign inadequate management of plastic scraps originating from the Netherlands. We therefore combine both methods to compare how much plastic waste enters the environment relative to the reported (known) plastic waste that is generated and collected in the Netherlands.

## Methods

### General setup

**Figure 1.**
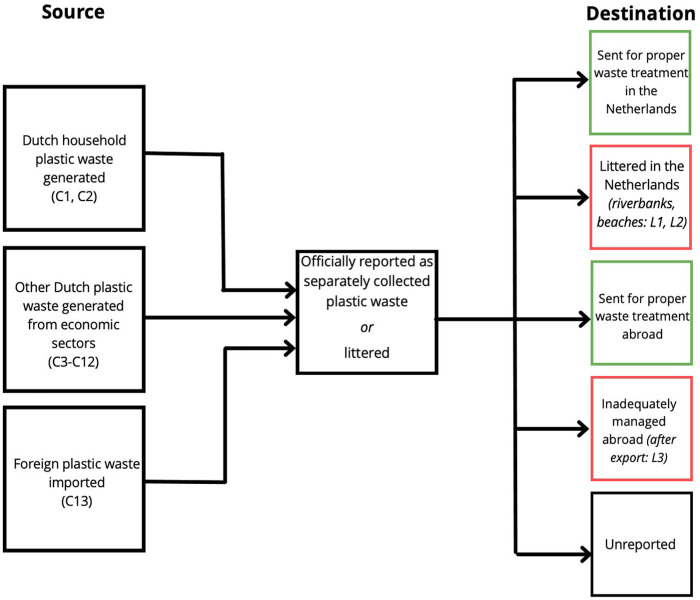
A schematic of the scope of our study. The left boxes represent the sources of our flow analysis with Dutch plastic waste generation from households (C1–C2 in Table 3), from other waste streams (C3–C12), or imported (C13). These flows are summed to estimate the total known (officially reported) waste that is separately collected or littered (central box). The right boxes represent the destinations of Dutch plastic waste: proper management and mismanagement in the Netherlands and abroad. Green boxes are Method 1 results (MFA), red boxes are Method 2 results (mismanagement model; L1–L3). ‘Unreported’ is for when the destination of a waste flow is not known or reported.

In our scope (represented in Figure 1), the first method uses the MFA approach (Brunner and Rechberger, 2016). The aim of an MFA is to create a systematic assessment of the sources and destination of a material (plastic waste, in our case) from a defined space (the Netherlands) and over a defined time (2017, as much as possible). For further information on the theory of an MFA, see Brunner and Rechberger (2016). The MFA ‘sources’ are Dutch plastic waste generated, collected and reported which we group into 13 categories. The ‘destinations’ are where the waste is sent to be processed (if known and reported). The boundaries of our flow analysis mean that we do not address the mass of plastic prior to our source (i.e. brought to the market or plastics still in use), nor do we address mass flows after our destination (i.e. new products produced by recycled plastics). Although imported waste does not originate in the Netherlands, once the waste enters the country, we assume it is ‘Dutch waste’; thus, it is part of our scope. We also define the uncertainty of each MFA source and destination to provide high, low and average estimates (section ‘Data uncertainties for MFA source categories (C1–C13)’).

The second method has been designed via a combination of previous data sets and models, hereafter called the mismanagement model. It is based on as much observational plastic litter data as possible that are applicable to our analysis (namely litter on Dutch beaches and riverbanks, following Kaandorp et al., 2022; van Emmerik et al., 2020, respectively). The ‘destinations’ are therefore measured Dutch littered plastics and inadequately managed plastics abroad (Figure 1). We include the latter to estimate when exported Dutch plastic scraps and waste (estimated in the MFA) are not properly managed in importing countries (using an adaptation of the method in Law et al., 2020). The term ‘unreported’ is used for when a plastic waste source or destination is not known or reported. To clarify, Methods 1 and 2 are therefore not simply two different ways to estimate the same result, they are complementary calculations to obtain one final overview of Dutch plastic waste flows.

### Method 1: Material flow analysis

#### MFA sources

Our 13 source categories (C1–C13) cover plastic waste generated by consumers and industry including households, manufacturing, construction and demolition, agriculture, electronics, vehicles and imports (Table 3). These sectors account for the major plastic waste origins in the Dutch economy (Plastics Europe, 2020; Snijder and Nusselder, 2019). The first nine categories (C1–C9) use the European Parliament and Council ‘Waste Generation’ classification, covering all Dutch NACE (Nomenclature of Economic Activities) codes (found in Annex 1, Section 8 of Regulation (EC) No. 2150/2002 and Supplemental Table S7). This is also the classification used by RWS Environment, which is the primary data source for our study (see section ‘Data sources for MFA waste streams’). The remaining four categories (C10–C13) are based on data we acquired from publicly available reports and statistical databases where possible and otherwise from expert judgement during personal communication. The detailed explanations and estimations of each MFA source are found in Supplemental Materials S1–S7.

#### MFA destinations

The endpoint of our flow analysis is the mass of plastic waste sent for (1) recycling, (2) incineration with energy recovery, (3) controlled landfilling, (4) reuse, (5) export (without specifying foreign waste management), (6) foreign proper waste management and (7) unreported. In the Netherlands, separately collected plastic waste sent to be incinerated is almost always with energy recovery (apart from 1 tonne of construction and demolition waste sent for incineration without energy recovery in 2017 (RWS database), hence its exclusion from our scope). Following the MFA theory of conservation of matter, the mass balance can be used to estimate the unexplained remainder (Brunner and Rechberger, 2016), which we classify as destination (7): unreported. To clarify, we do not assume that unreported is indicative of ‘mismanagement’ since we assign high uncertainty to some categories, such as the destination of imported scraps (100% uncertainty; Table 1), which are highly unlikely to all end up in the environment. Without any indication of the fraction of unreported waste ending up as mismanaged, we have decided against any assumptions and instead include lower and upper mismanagement estimates that may account for such discrepancies (Supplemental Table S1). We also account for the destination of plastic waste that was originally sent to Dutch recycling plants but then sent to controlled landfills in the Netherlands or to foreign recycling plants (reported by the Dutch Ministry of Human Environment and Transport Inspectorate (Inspectie Leefomgeving en Transport), hereafter ILT; Overheid: Ministerie van Infrastructuur en Waterstaat, 2021). Uncontrolled landfills do not occur in the Netherlands and hence they are not a destination in our MFA.

**Table 1.** Estimation of data uncertainties for the MFA sources and destinations and the main causes of the uncertainties.

Source categories (C1–C13)	Data uncertainty for sources (%)	Causes of source uncertainties	Data uncertainty for destination (%)	Causes of destination uncertainty	Data sources
Household plastic packaging (C1) non-packaging plastic waste (C2)	±5	Statistical errors	±10	Statistical errors	RWS database: based on CBS data, surveys and interviews, 2022
Separately collected plastic waste (C3–C9)	±5	Statistical errors and the estimate of non-plastic contamination.	±10	Statistical errors and the estimate of non-plastic contamination.	RWS database: based on CBS data, surveys and interviews, 2022
End-of-life vehicles (C10)	±10	Data provided by personal communication (not yet published); however, the Dutch government Inspection Authority collected their data. Equivalent to official database.	±10	Estimates of fraction of plastics assumed based on average weight (1035 kg) but can be up to 3000 kg. Estimates of ELVs sent for export have a negligible uncertainty	Personal communication with ARN and ILT and CBS (exports), 2022
E-waste (C11)	±10	Statistical error	±50	The destination of the plastic fraction in e-waste is not reported.	Source: Global E-waste monitor (Forti et al., 2020), ProSUM project (Huisman et al., 2017. Destination: Personal communication with Stichting OPEN
Textiles and clothing (C12)	±20	Fraction of synthetic (plastic) fibres in textile products per country is not reported. We use global fibre estimates (63%). Data also for 2018, not 2017 (The Fiber Year, 2019).	±50	The destination of the plastic fraction in textiles is not reported.	National survey (Hopstaken et al., 2020)
Imported plastic scraps (C13)	Negligible	Registered imported amount via CBS and UN Comtrade has negligible uncertainty	±100	Unreported destinations of all traded waste. Unknown how much imported plastic scraps were registered using Rotterdam as a transit harbour for further exporting or were recycled in the NL.	Trade statistics (UN Comtrade) and national statistics (CBS), personal communication, with ILT and NRK, 2021
Unreported (source for exported waste)	Negligible	Registered exported amount via CBS and UN Comtrade has negligible uncertainty	NA	We account for uncertainties and ranges in the mismanagement model	Trade statistics (UN Comtrade)

CBS: Central Bureau of Statistics; RWS: Rijkswaterstaat; ARN: Auto Recycling Nederland; ILT: Inspectie Leefomgeving en Transport (Human Environment and Transport Inspectorate); NRK: Federatie Nederlandse Rubber en Kunststofindustrie (Federation of Dutch Rubber and Plastics Industry); Stichting OPEN: OPEN Foundation; NL: Netherlands; ELV: end-of-life vehicles.

#### Data sources for MFA waste streams

RWS environment is responsible for the data collection of Dutch waste flow quantities (Rijkswaterstaat: Environment, 2021). They collaborate with other monitoring organisations to gather and analyse the data for their central database (hereafter, RWS database). The data include waste generated, collected and where it is sent to be treated or processed (our categories C1–C9). Since these data are used for policymaking by the Dutch government and for other official statistical obligations (e.g. Eurostat), we use the RWS database as much as possible and assign a very low uncertainty (Table 1). Although the raw data are not publicly available online, they can be requested for research purposes, as was the case for this study during interviews. Their data include household and separately collected waste (hence, excluding non-household municipal solid waste plastics, see below). We chose to use 2017 data since it is the latest, most accurate data; it can take several years for RWS to gather the data from all waste sectors and to generate reliable waste statistics that meet (inter)national standards. For example, the composition of household municipal solid waste plastics represents an average over 3 years; hence, 2017 data are an average of 2016–2018 quantities (Rijkswaterstaat, 2021). We base our other category estimates C10–C12 on data from reports and interviews (see Table 1 and Supplemental Materials S4–S6). For imported plastic waste (C13), we use the United Nations Comtrade database (United Nations, 2022), commodity code: 3915 (waste, parings and scrap, of plastic) which we hereafter refer to as ‘plastic scraps’. The UN Comtrade is an open repository that provides official data on international trade of goods between 170 countries and is maintained by the United Nations Statistics Division. The Netherlands provides their annual trade statistics to the UN Comtrade via Statistics Netherlands (CBS).

#### Beyond our scope

As mentioned above, RWS (and the CBS) have data on post-consumer and post-industrial plastic waste that is collected by municipalities: (1) mixed municipal solid waste (MSW) from households and (2) separately collected waste streams (when plastics are disposed of in a separate container by households or other sectors; see Supplemental Material S1 for further details). Non-household sectors also generate mixed waste that is not separately collected, for example, mixed waste from railway stations and offices. Here we call this stream ‘mixed waste from non-household sectors’, which is similar to the waste defined in Brouwer et al. (2020) as ‘other post-industrial plastic packaging waste’. This waste is not collected by municipalities but by individual private organisations or companies (RWS, personal communication, 2022). Without a centralised data monitoring system to track the quantity, composition or destinations, we cannot include this waste stream in our scope. Brouwer et al. (2020) also state that this post-industrial mixed waste is heterogeneous (composed of many types of plastics) and reports to estimate the composition or purity of the polymers are lacking. Although their model assumes that the composition is similar to post-consumer waste, we exclude this waste stream; we instead provide suggestions that this ‘unknown’ should be monitored more closely in the future.

Since the data we use are not always classified by polymer type, our results represent a mixture of different polymers. This is not of concern to us since our goal is to determine the total plastic waste mass budget (however, future work exploring degradation in the environment or circularity of specific plastics, e.g. different polymers would need to be investigated).

There are other hidden plastic waste streams in traded goods, such as plastics found in refuse derived fuel (RDF). In 2016, the Netherlands imported approximately 1.2 million metric tonnes of RDF (Rijkswaterstaat, 2021). Although RDF could contain as much as 40–50% plastics (RWS, personal communication, 2022), without any credible data sources and without them being traded as ‘plastic scraps’ we exclude them from our MFA.

#### Data uncertainties for MFA source categories (C1–C13)

Table 1 shows an overview of the data uncertainties identified in this study. We consider low uncertainty (±5%) for primary data obtained from national statistics, well-documented national surveys and industrial data or personal communications with possibilities for verification and validation (e.g. independent scientific papers, expert judgement, industrial association surveys). For example, the source data for separately collected plastic waste from the RWS database in the streams of C1–C9 have a 5% uncertainty. For data originating from documented national or regional surveys, with few independent sources to validate, that strongly rely on expert judgements or assumptions (e.g. the source data of C12, textile and clothing), we assign a medium uncertainty of ±20%. For data largely based on assumptions and/or highly aggregated statistics with little verification, we assign a high uncertainty, ±50%. This holds true for the destination data for e-waste (or waste from electrical and electronic equipment (WEEE): C11) and textile waste (C12) due to unknown management of the plastic fraction (see Supplemental Materials S5 and S6; Supplemental Tables S3 and S4). For the destination data of imported plastic scraps (C13), we assign a 100% uncertainty since we only have one expert judgement (and it cannot be independently verified). We use these uncertainty values to generate upper and lower bounds around the average estimate; for example, for low uncertainty data, the upper estimate is the average plus 5%, and the lower estimate is the average minus 5% (displayed in Table 3). For further details on the data sources and waste categories, see the Supplemental Materials S1–S7.

### Method 2: Mismanagement model scope

Method 2 (the mismanagement model) is designed to estimate the mass of Dutch plastic waste (from known sources) ending up in the environment. Our model is based on three data sets: plastic litter data collected on Dutch riverbanks (L1) and Dutch beaches (L2), and estimated Dutch plastic scraps entering foreign environments due to inadequate management in importing countries (L3). To use discrete field data samples (from site locations not biased towards relatively polluted measurement locations) for our analysis, we assume that all items were removed after each sampling event. By using the days between the sampling events, we can calculate the deposition of items per day and hence per year. We extrapolate these results temporally and spatially to provide annual national estimates of plastic waste littering rates on beaches and riverbanks. Since these data are scarce, we decide to use data from all years available to estimate an annual flux, instead of only 2017 data. It must be noted that these samples are standing stocks (a sample of the total amount at a particular point in time) and since rivers and coastlines are dynamic systems, the daily fluxes could fluctuate, which is not represented in these results. See Table 2 for all definitions used in this study for the mismanagement model.

**Table 2.** Defined terms in the study for mismanaged Dutch plastic waste, following the Jambeck et al. (2015) and Law et al. (2020) definitions.

Terms used in this study	Definition
Litter	Solid waste that is intentionally or unintentionally disposed into the environment despite the availability of waste management infrastructure (in this study, using estimates of litter on Dutch riverbanks and beaches).
Inadequately managed waste	Solid waste that is not collected and/or properly contained because of lack of waste management infrastructure (waste reported in ‘open dump’, ‘waterways’, ‘unaccounted for’ and ‘other’ categories in Kaza et al., 2018).
Exported, inadequately managed plastic scraps	Plastic waste collected for recycling in the Netherlands that was exported to countries where it was inadequately managed and assumed to end up in the environment in the importing country.
Mismanaged waste	Sum of above categories (all known and estimated Dutch plastic waste entering the environment in the Netherlands and abroad).

#### Riverbank estimates (L1)

We use data from van Emmerik et al. (2020) which were collected up to twice a year on the riverbanks of the Dutch Meuse and Rhine rivers from 2017 to 2019. Their litter items were classified following the OSPAR (Oslo and Paris Conventions) Commission protocol which we re-categorised into our 13 MFA categories (see Supplemental Material S9 and Supplemental Table S5b for more details). Note that since the Rhine flows through Switzerland, France and Germany and the Meuse flows through France and Belgium, the litter found on Dutch riverbanks may also be foreign waste; however, just as we include imported waste in our MFA scope, once waste reaches Dutch riverbanks we define it is as Dutch litter.

Since the van Emmerik et al. (2020) method does not include the mass of sampled litter items, we use van Emmerik and de Lange’s (2021) riverbank mean item mass estimates for each of our 13 categories (see Supplemental Material S9 for more details on van Emmerik and de Lange, 2021). By multiplying the number of items by the average mass, a total mass per category per sampling length (100 m) per year is estimated. Standard deviations (per item within each category) are used to provide a range around the mean estimates, which result in being up to one or two orders of magnitude (Supplemental Table S5a). We extrapolate spatially across the Netherlands using the length of all major rivers in the Netherlands from RWS (Rijkswaterstaat Rivers, 2022). The total plastic mass on Dutch riverbanks *P*_r_ (kg) is therefore estimated as:

(1) $$P_{r}=L_{r}10\rho_{p,r},$$

(2) $$\rho_{p,r}=\sum_{i=1}^{13}I_{i,r}\bar{m_{i,r}},$$

with the total length of Dutch rivers *L*_r_ (908 km), total plastic mass density *ρ*_p,r_ (kg/100 m; kilograms per 100 metre sampling length), unit correction 10, item density *I*_*i*,r_ (items/100 m) per item category *i* and mean mass $\bar{m_{i,r}}$ per item category (kg/item) *i*. We then estimate the total plastic density mass by summing all 13 categories. For the upper estimate, we sum the standard deviation to the $\bar{m_{i,r}}$ , producing an order of magnitude larger than the average for almost all categories. For the lower estimate, we therefore subtract an order of magnitude from the average for each category. See further details in Supplemental Table S5a.

#### Beached estimates (L2)

Kaandorp et al. (2022) developed a model to estimate beached litter quantities, based on data collected along the Dutch North Sea coastline by volunteers from the North Sea Foundation during the month of August for six consecutive years (2014–2019). As the beach litter was collected only once per year per location as opposed to twice a year for the riverbanks, beached litter fluxes could be underestimated compared to the riverbank litter fluxes since a higher litter collection frequency results in higher flux estimates (Ryan et al., 2014). Furthermore, as with the riverbank data, we cannot take into account whether the plastic litter originated from the Netherlands only.

The volunteers weighed the total litter collected and identified the items (wherever possible) and found that the overall plastic fraction of the litter in terms of numbers is 80–90% (Kaandorp et al., 2022). This matches recent findings from Scottish beached OSPAR data (Smith and Turrell, 2021) (88.3% in terms of numbers, 86.6% in terms of weight). The regression model in Kaandorp et al. (2022) provides results as a minimum and maximum yearly standing stock of total litter (or annual net flux, in our case), based on 95% confidence intervals. We therefore estimate the minimum and maximum beached plastic flux in terms of weight by taking 86.6% of the modelled minimum and maximum flux of total litter.

#### Inadequate management after export of plastic scraps and separately collected waste (L3)

We follow the method developed in Law et al. (2020) to estimate inadequate management of exported Dutch plastic scraps in importing countries. We first extract the list of countries that imported Dutch plastic scraps in 2017 from the UN Comtrade database: 383.4 kt in total was sent to 56 countries in 2017 (see Supplemental Table S6a). From these 56 countries, we identify 16 countries as having a national inadequate waste management above 20% in 2017; ‘inadequate waste management’ refers to total local MSW sent to uncontrolled landfills, open dumps, waterways, etc., as published in the World Bank report (Kaza et al., 2018). We assume that the UN Comtrade database includes all registered traded plastic scrap amounts from the largest Dutch trading companies (via CBS reports). The Netherlands enforces tight regulations on trade of waste where only scraps that fulfil the conditions of the Greenlist categories of the European Waste Shipment Regulation (EWSR; 1013/2006 EU) may be exported without a notification sent to Dutch authorities (the ILT). This ensures that all exported plastic scraps only consist of high quality, recyclable material and exported bales can only have a maximum non-plastic contamination (e.g. sludge, dirt) of 2% in mass (Overheid, 2022). The enforcement of these laws is very strict, where suspects can be prosecuted by criminal law (de Paauw Recycling B.V., personal communication, 2022).

The amount of recyclable exported Dutch plastic scraps that was actually recycled in importing countries in 2017, however, was not reported due to a lack of formal external audits. Nowadays, such audits are starting to be implemented and once they become publicly available, ground-truthing of estimates of recycled amounts of exported plastic scraps will be possible. We therefore use insights from reports (UTS, 2020) and our background interviews with Dutch traders to deduce estimates for our mismanagement model (with high levels of uncertainty). Firstly, at any recycling facility, regardless of the country, bales of fully ‘recyclable plastic’ can result in a yield loss of 5–15%, due, for example, to contamination or plastic pieces being too small to recycle (Kras Recycling B.V., personal communication, 2023). Taking into account the legal 2% maximum of non-plastic contamination discussed above, 3–13% of the mass can be plastic scraps that are rejected during recycling. Since ‘yield loss’ results in financial loss if not used, recyclers strive to convert the fine pieces into agglomerates for raw material (to produce pipes or bricks, e.g. Kras Recycling B.V., personal communication, 2022). Some of the large trading companies in the Netherlands also have signed documents from the companies importing their waste stating that the importers are obliged to prevent harm to the environment (de Paauw Recycling B.V., personal communication, 2022). Though less plastic is expected to be at risk of ending up in the environment by larger recycling facilities in importing countries (with the right equipment and regulations), it might be harder to track unregulated informal recycling techniques and to track the fate of scraps from Dutch traders that export smaller amounts, hence the need for audits of all traded plastic scraps (which our interviewees also endorsed). It is well known that countries with inadequate waste management systems struggle to manage their own domestic plastic, hence importing waste adds pressure to facilities running over capacity (Lau et al., 2020; Liang et al., 2021). Furthermore, in the case of Vietnam, for example, 90% of imported plastics are recycled by informal small-scale businesses, increasing the risk of smaller pieces being missed during manual detection or detection by old, low-tech equipment. Vietnam is ranked third highest for Dutch imports out of the 16 countries with inadequate waste management above 20%; for more information on Vietnam’s recycling, see Supplemental Material S10 and UTS (2020).

With these combined considerations, we assume that of the 3–13% rejected by recycling methods in importing countries, 25–75% is at risk of being mismanaged. This results in 0.75–9.75% of total exported plastic scraps at risk of entering the environment (or open dumps) in countries known to have inadequate waste management of more than 20%. This is a modification of the Law et al.’s (2020) method (estimated that 15–25% exported US waste was non-recyclable), since the United States does not have a strict notification and Greenlist system like the Netherlands. These steps are shown in detail in Supplemental Table S6a and explained in Supplemental Material S10.

Another means for Dutch plastic waste to end up in foreign territory is after export of second-hand products for which the final destination is not reported, from textiles and clothing, ELVs and e-waste. These amounts are included in the ‘Mismanaged’ category (Table 3). Following the method in Law et al. (2020), assuming that these items are of very low quality and (since they are not reported as being sent for proper waste management) are rejected from waste treatment plants, 25–75% are at risk of entering the environment. Therefore, 25% is used for our low estimates of inadequate plastic scraps management, 50% for our average estimates and 75% for our high estimates of inadequate plastic scraps management. See Supplemental Table S6b for more information. The initial source of exported Dutch waste is not provided by the UN Comtrade or other centralised reports; it is unknown how much is waste generated in the Netherlands or foreign waste that was imported and then re-exported. Although it is suggested that the imports entering the large port of Rotterdam are mostly re-exported (European Environment Agency, 2021), without reported quantities, we have been advised to classify the source of exported Dutch waste as ‘unreported’ (ILT and RWS, personal communication, 2021). This ‘unreported’ source does not contribute to the total Dutch plastic waste generated, to avoid double-counting (e.g. if it comes from separately collected waste or imported waste).

**Table 3.** Results from Method 1 (the MFA: categories C1–C13) and Method 2 (the mismanagement model: categories L1-L3).

Category	% of total waste generated	Waste generated (kt)	% Sent to recycling	Sent to recycling (kt)	% Sent for energy recovery	Sent for energy recovery (kt)	% Sent to landfill	Sent to landfill (kt)	% Sent to be reused	Sent to be reused (kt)	% Sent for export	Sent for export (kt)	% Sent to waste plant abroad	Sent to waste plant abroad (kt)	% Unreported	Unreported (kt)	Mismanaged (kt)
Imported plastic scraps (C13)	31	623	16	100 (±100)	0	0	0	0	0	0	0	0	0	0	6	523 (423–623)	0
Household plastic packaging (C1)	20	398 (±40)	43	169 (±17)	57	229 (±23)	0	0	0	0	0	0	0	0	0	0 (0–20)	0
Textiles and clothing (C12)	13	250 (±50)	1	2 (±1)	48	121 (±60)	0	0	3	7 (±3)	2	6 (3)	44	109 (±54)	2	5 (0–77)	3 (1.5–4.5)
Household plastic non-packaging (C2)	10	203 (±20)	14	28 (±3)	72	146 (±15)	2	4 (±0)	0	0	0	0	0	0	12	25 (18–33)	0
Industry manufacturing (C4)	8	157 (±16)	90	141 (±14)	9	14 (±1)	1	2 (±0)	0	0	0	0	0	0	0	0	0
Transport storage (C6)	5	103 (±10)	62	64 (±6)	5	5 (±1)	0	0	32	33 (±3)	0	0	0	0	0	0 (0–5)	0
Services administration (C5)	5	91 (±9)	70	64 (±6)	15	13 (±1)	15	14 (±1)	0	0	0	0	0	0	0	0	0
E-waste (C11)	4	75 (±8)	38	29 (±14)	20	15 (±8)	1	0	0	0	3	2 (±1)	8	6 (±3)	30	23 (4–41)	1 (0.5–1.5)
End-of-life vehicles (C10)	2	35 (±11)	13	5 (±2)	66	24 (±2)	0	0	0	0	21	7 (±7)	0	0 (±7)	0	0 (0–1)	3 (1.5–4.6)
Agriculture, forestry and Fishing (C7)	1	30 (±3)	78	23 (±2)	0	0	22	7 (±1)	0	0	0	0	0	0	0	0	0
Building construction (C3)	1	21 (±2)	90	19 (±2)	0	0	10	2 (±0)	0	0	0	0	0	0	0	0	0
Water treatment supply (C8)	0.08	2 (±0)	100	2 (±0)	0	0	0	0	0	0	0	0	0	0	0	0	0
Energy and mining (C9)	0.06	1 (±0)	92	1 (±0)	8	0.1 (±0)	0	0	0	0	0	0	0	0	0	0	0
Littered (L1 and L2)	0.01	0.1 (0.02–0.9)	0	0	0	0	0	0	0	0	0	0	0	0	0	0	0.1 (0.02–0.87)
Unreported (source for L3)	NA	305	0	0	0	0	0	0	0	0	0	305	0	0	0	0	5.3 (0.7–9.8)
From recycling	NA	NA	0	0	0	0	5	39	0	0	0	0	10	78	0	0	0
TOTAL		1990 (±111)	33	647.8 (±167)	29	567.1 (±111)	3	28.3 (±3)	2	40.1 (±7)	16	320.3 (±5)	10	193.5 (±58)	5	575.8 (445– 801)	12.4 (4.3–21.2)

Macroplastic waste source categories and destinations within and outside the Netherlands in 2017 are shown. Values within brackets represent error (low and high estimates). Rows are sorted from categories with highest to lowest % of total waste generated. The last two rows do not contribute to total waste generated to avoid double-counting. See Supplemental Material S1 for more details and to see results from the methods separately.

#### Beyond our scope

Annual, national estimates of land litter are still unknown (reasons for this are explained in section ‘Discussion’), so our litter estimates should be considered a lower estimate. Also beyond our scope is microplastic litter (such as abrasion from tyres or microfibres from clothing), which is the focus of many other studies (e.g. Besseling et al., 2014, Mani et al., 2015, Mintenig et al., 2020). Although microplastics can have devastating impacts on the environment, their effect on the mass balance of plastic waste is likely minimal (Lebreton et al., 2018, Ryan et al., 2020). To remain consistent with the type of riverbank environment sampled, all lakes, large estuaries, canals and other bodies of water less than 30 m wide are excluded from our study. Furthermore, we do not include estimates that have flowed into (and stay in) the ocean from riverbanks or beaches. Although we know that not all plastics from riverbanks and beaches will end up in the North Sea, to prevent estimating the fraction that does and potentially double-counting, we do not include them in our analysis. Our estimates could also be under-representations due to removal of litter by other beach or riverbank clean-up efforts and environmental factors (wind or rain). Our uncertainty ranges can partially account for some these factors (Supplemental Table S1).

## Results

### MFA outcomes

Our MFA results suggest that 1990 (±111) kt of Dutch plastic waste was generated in 2017 (from known, reported estimates), where ‘generated’ includes both plastic waste generated within the Netherlands and imported into the Netherlands. About 31% of the 1990 kt is estimated to have come from imported plastic scraps (623 kt; United Nations, 2022); equivalent to almost half of the plastic waste generated directly in the Netherlands (1367 ± 111 kt). This is comparable to the Law et al.’s (2020) estimate of 1251 kt of Dutch plastic waste generated, using data from 2015 (Kaza et al., 2018). We therefore estimate that 80 kg of plastic waste was generated per person in the Netherlands in 2017 (using the total of 1367 kt and the Dutch population estimate of 17,131,296 in 2017; The World Bank, 2022). We do suggest, however, that our analysis underestimates the total since the plastic within mixed waste from non-household sectors are not reported and therefore cannot be included (see section ‘Beyond our scope’).

**Figure 2.**
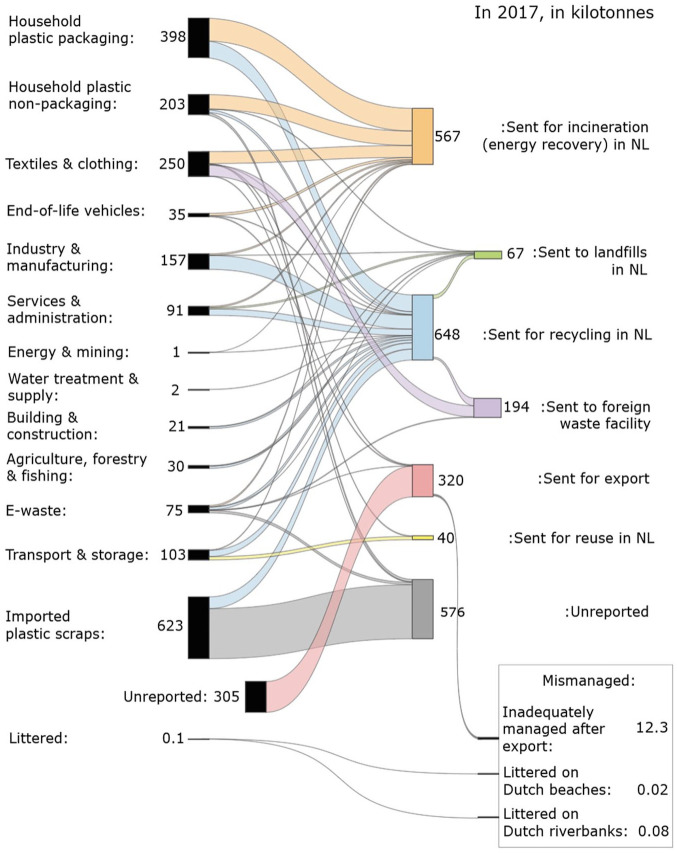
Sankey diagram summarising the results from Method 1 (MFA) and Method 2 (the mismanagement model). On the left are the sources of Dutch plastic waste generated in 2017 (in kt) and on the right are the destinations. The values shown are the average estimates. Ranges for the low and high estimates (representing the error bars) are found in Table 3 and Supplemental Table S1a–c.

Of the categories we include in the MFA, the highest contributors to the total plastic waste generated in the Netherlands were household plastic packaging (HPPW; 398 kt; which is equivalent to 20% of the total plastic waste generated, 1990 kt total), textiles and clothing (250 kt; 13% of the total), and household plastic non-packaging (203 kt; 10%); Table 3. All other categories were below 10% of the total. Of the 1990 kt, we estimate that 88% was sent to three destinations: 33% (648 kt) was sent to be recycled in the Netherlands, 29% (567 kt) was sent to be incinerated for energy recovery in the Netherlands and 26% (514 kt) was sent for export (Table 3 and Figure 2). The rest was sent to controlled landfills (for non-shreddable or non-combustible plastic waste), for reuse within the Netherlands, or unreported. The three waste flows originating from Dutch waste with unreported amounts sum up to 53 kt; household plastic non-packaging (25 kt), e-waste (23 kt) and textiles and clothing (5 kt). As mentioned above, following expert judgement, we do not assume that ‘unreported’ represents mismanagement (RWS and Stichting OPEN, personal communication, 2021).

One of the categories that we want to highlight is HPPW. We suggest that in 2017, 398 kt of HPPW was collected, where 173 kt came from PMD (plastics, metal and drink/beverage cartons), 17 kt was separately collected and 208 kt was within mixed MSW. These results are mostly net masses (after accounting for residual contamination), the calculations of which are explained in the Supplemental Material S1 and Supplemental Table S2a. An estimate of 42.5% of HPPW was sent to be recycled and 57.5% was sent to be incinerated for energy recovery. The most comparable study concerning HPPW in 2017 (Brouwer et al., 2019) estimated that 350 kt of HPPW was collected, approximately 38.5% of which was sent for recycling and 61.5% of which was sent for incineration. The slight differences between our results could be due to different scopes, since they use some estimates based on data from earlier years when HPPW was still divided into PMD, PM (plastics and metal) and P (plastics). Furthermore, they report recycled amounts in terms of dried washed milled goods, so non-targeted packaging components that are also removed during recycling are subtracted (Brouwer et al., 2019).

### Mismanagement model outcomes

**Figure 3.**
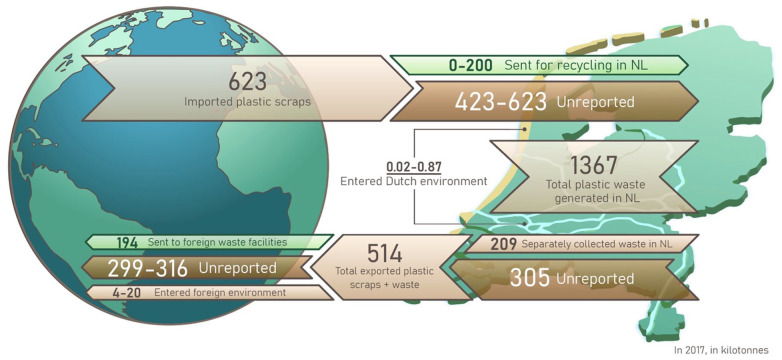
An overview of our main results (in 2017, in kt): (1) total plastic waste generated within the Netherlands (without imported scraps): 1367 kt, (2) the destination of imported plastic scraps (623 kt): mostly unreported, (3) the source and destination of exported plastic scraps and waste (514 kt): mostly unreported, (4) foreign inadequate management abroad after export (4–20 kt) was much larger than domestic littering (0.02–0.87 kt on riverbanks and beaches). Domestic estimates do not include land littering so are most likely underestimated. Source: Image created by thisillustrations.com.

We estimate that 12.4 (4.3–21.2) kt of Dutch plastics in 2017 reached Dutch riverbanks, Dutch beaches and the environment in foreign territory after being imported. This is equivalent to approximately 0.7 kg (on average)/person/year, or about 145 items (if an average item has a mass of 5 g; van Emmerik et al., 2020). Since we only consider littered estimates on Dutch beaches and riverbanks, 0.02–0.87 kt/year (Figure 3) is likely an underestimation of plastic entering the Dutch environment. Previous Dutch littering estimates such as 27.7 kt by Jambeck et al. (2015) in 2010 could be an overestimation; Ryan et al. (2020) and Chitaka and von Blottnitz (2019) suggest that the Jambeck et al. (2015) solid waste inadequate mismanagement in South Africa is overestimated by roughly an order of magnitude. Estimates for Dutch land-based plastic littering are currently unknown and are required to estimate the total Dutch littering rate.

We estimate that 81 (8–843) tonnes of plastic waste (0.2–4.0% of the total mismanagement) end up on Dutch riverbanks annually; the standard deviation spans two orders of magnitude due to the extrapolation based on only a few annual samples. Our average estimate is within the range of previous estimates of plastic macrolitter entering the ocean from Dutch rivers: 41–271 tonnes (González-Fernández et al., 2021; Meijer et al., 2021; van Emmerik et al., 2020;) and from the most recent study based on river transport observations: 120 tonnes (van Emmerik et al., 2022). We acknowledge, however, that not all litter on riverbanks ends up in rivers and flows into the North Sea (see section ‘Beyond our scope’).

We estimate that 21 (14–27, 95% confidence interval) tonnes of plastic waste (0.1–0.3% of the total mismanagement) end up on beaches per year in the Netherlands. Caution is advised when directly comparing the beached estimates and riverbank estimates since the sampling frequency was different (yearly for beaches and up to every 6 months for riverbanks; see Ryan et al., 2014). The standing stock of plastic waste on beaches varies substantially by about 600 kg/day on average over the entire coastline (Kaandorp et al., 2022b).

It could therefore be that the yearly estimates are in fact similar for both riverbanks and beaches (somewhat accounted for by our uncertainty ranges). Our results could also be supporting the hypothesis that plastic litter on riverbanks can be retained there for years, decades or potentially longer (van Emmerik et al., 2022). European countries export a large amount of plastic waste (4.3 million metric tonnes in 2017; UN Comtrade) due to insufficient domestic capacity to process the waste or cheaper foreign processing costs (Inspectie Leefomgeving en Transport, 2021). Using our model as a first-order estimate, 0.7–9.8 kt of exported Dutch plastic scraps could end up in the environment of non-Dutch territory. This mass is equivalent to 0.2–2.6% of exported Dutch plastic waste (383 kt) in 2017, which is similar to Bishop et al.’s (2020) findings. They estimate that on average 3% of exported European polyethylene waste (or 4.8 kt from the Netherlands in 2017) ended up in the (global) ocean (Gradus, 2020). Furthermore, we do not include illegal or unregistered smaller trading companies that are not in the UN Comtrade database which could export plastic scraps with higher fractions of non-recyclable material (de Paauw Recycling B.V. and Kras Recycling, personal communication, 2023).

By including inadequate management of exported second-hand items, estimates of plastic scraps entering the foreign environment reach 4.3–20.4 kt (Figure 3), which represents 0.2–1.0% of the total amount of Dutch plastic waste generated (1879–2101 kt). This compares to the lower bound of the US estimates from Law et al. (2020), where 0.4–2.4% of the total US plastic waste generated (42,000 kt) entered the environment. The upper estimate could be higher for the United States, both because the amount of exported waste (as a fraction of total generation) was higher, or because the exported waste in 2017 was sent to more countries with >20% inadequate management. The Chinese plastic waste import ban went into effect in 2018 and one could expect plastic waste export to have decreased since 2017 as it did for many other counties (Wen et al., 2021). However, recomputing our mismanagement model based on the latest UN Comtrade data (in 2020), Dutch plastic scraps entering the foreign environment increase by 28.7% (to 1.0–12.6 kt; Supplemental Table S6c) in 2020 relative to 2017. There could, however, be a decrease in these estimates in the future if we take the predicted growth in domestic recycling capacity into account, following the Plastic Pact (Plastic Pact NL, 2021; van Veldhoven- van der Meer, 2019).

We estimate that the mass of exported Dutch plastic scraps ending up in the foreign environment is up to 1000 times larger than on Dutch riverbanks and beaches. Due to the large mass of plastic scrap exported, even a small inadequate management rate results in a relatively large estimate entering the environment. This highlights that when trying to understand the impact of plastic pollution, tracing plastic waste pathways beyond one’s borders is essential. An important caveat is that for the Dutch inadequate management, only riverbank and beached plastic litter is included, whereas the exported waste covers all mismanaged plastic scraps, regardless of end fate location. For example, much of the non-recyclable plastic scraps from exports might end up in dumpsites; although this waste is not controlled in the same manner as in European sanitary landfills, it is still more controlled than that found on riverbanks and the coast in the Netherlands. Hence, caution should be taken when comparing the estimates of plastic ending up in the Dutch environment and abroad. Furthermore, as seen from Figure 3, our analysis highlights the unknowns resulting from lack of reporting of sources and destinations of traded plastic scraps and waste. This is therefore also one of our key recommendations when filling data gaps.

## Discussion: Recommendations for the unknowns

The Dutch authorities have extensive data and reports for many plastic waste streams, more than some countries, such as the United States (Law et al., 2020), but less than other countries, such as Switzerland (Kawecki et al., 2018). However, unknowns and uncertainties have become evident during our study, so we highlight five main recommendations to attain more complete overviews of plastic waste flows in the Netherlands (and other high-income countries) in the future. Before expanding on the five recommendations below, one aim for policymakers should be to oblige all waste reporters to provide timely data so the most reliable statistics are generated sooner than 5 years later.

### Tracking the source and destination of traded plastic scraps

To our knowledge, there is no publicly available, centralised report of the amounts of traded plastic scraps into and out of the Netherlands that are properly managed. Bishop et al. (2020) analyse trade of European polyethylene and assume that high-income countries recycle 90% of the waste. However, this is likely not the case in the Netherlands since according to expert judgement (ILT, personal communication, 2021), the Netherlands does not have the capacity to recycle an extra 623 kt of plastic scraps (since the maximum capacity is closer to 200 kt) and re-exporting is likely high (European Environment Agency, 2021). With the amount of imported plastic scraps (623 kt) equivalent to almost half of the total plastic waste generated within the Netherlands (1367 kt), reporting what happens to this waste is imperative. Furthermore, estimates of plastic scraps entering the environment after inadequate management in foreign territory would greatly be improved by directly tracking how much is recycled abroad. Countries also have different auditing regulations for imported waste (they accept different levels of contamination; Kras Recycling, personal communication, 2023) and recycling rates of imported (often higher quality) waste can be different to domestic waste recycling rates (e.g. in Indonesia; Darus et al., 2020). Further studies should include a country-by-country analysis that accounts for such differences to attain a clearer estimate of the mass of plastic in the environment.

Recent trade regulations linking plastic scrap trade and circular economy targets have been developed (outlined below) which, if enforced, can help to track and reduce foreign plastic waste inadequate management:

1. In January, 2021, 180 of the countries in the Basel Convention signed a new agreement, BC14-12 (Inspectie Leefomgeving en Transport, 2021) stating that only clean, sorted and recyclable plastic waste may be freely traded. Export of all other plastic waste (e.g. of mixtures of different plastics or contaminated waste) must be notified to the EWSR. Today, most of the Dutch registered plastic waste shipments should already be mono-polymers that are easily recyclable (ILT, personal communication, 2021), guaranteeing that new smaller businesses adhere to this (and illegal shipments are stopped) can facilitate proper foreign waste management.
2. The EU is revising its 2006 EWSR in favour of intra-EU trade. The European Environment Agency (2021) report shows that intra-EU shipment of good quality, recyclable waste follow principles of a circular economy for numerous reasons: (1) developing the ‘economies of scale’, meaning the cost of waste treatment and secondary raw materials reduces; (2) improving security of supply for recycling facilities, with a steady flow of secondary raw materials for producers; (3) developing technologically advanced (and low carbon emitting) facilities within this economically competitive business model (European Environment Agency, 2021).
3. As of January 2020, the Netherlands has started taxing all waste imports that are destined for incineration and landfills (van Veldhoven Van der Meer, 2021), which could see a reduction in imports into the Netherlands in the coming years.
4. Two separate pieces of EU legislation (Directive 2018/851/EG: The Waste Framework Directive adjustment and 2018/852/EG: The Packaging Directive adjustment) state that exported waste sent for recycling can only count towards attainment of targets ‘if the exporter can prove that the treatment of waste outside the Union took place in conditions that are broadly equivalent to the requirements of the relevant Union environmental law’ (Directorate-General for Environment et al., 2019). If auditing and reporting of foreign recyclate yield from imported Dutch plastic scraps is enforced, end-of-life traceability of exported Dutch plastic could be attained (and plastic entering the environment prevented). Although audits of exported material quality are strictly regulated by the large Dutch trading companies, these companies stress the need for legal enforcement to report how much exported material is actually recycled (de Paauw Recycling B.V. and Kras Recycling, personal communication, 2023). Recycling yield loss can be 5–15% in any recycling facility, where the residual loss could end up in landfills or incinerated (Kras Recycling, personal communication, 2023).

As well as enforcing the regulations above (and following other suggestions outlined in the report, UTS, 2020), we recommend to (1) increase domestic (and EU) recycling capacities, while reducing waste sent to incineration and landfills (van Veldhoven Van der Meer, 2021, Wijngaard et al., 2020); (2) prohibit untraced export of European waste outside the EU (as also suggested by Friant et al., 2022) or to any country with ‘inadequate waste management’ (i.e. more than 20%; Law et al., 2020); (3) link efforts to an already existing successful policy, such as the Extended Producer Responsibility, where plastic product producers could pay for and ensure proper waste management both within and outside the country of purchase.

### Reporting plastics in mixed waste from non-household sectors

Household plastic waste and separately collected waste from economic sectors (C1–C9) are closely monitored after collection in the Netherlands (Brouwer et al., 2019; Rijkswaterstaat, 2021). The missing data include the sources and destinations of plastics in mixed waste from non-household economic sectors, for example, post-consumer and post-industrial waste from stores, train stations and restaurants that are collected from private companies. We recommend to monitor these flows (e.g. by regular surveys coordinated at a national level) to determine the quantity, the composition and how this plastic waste is processed. Although some information could have been extracted from grey literature (e.g. PlasticsEurope), we could not use these data when the sources were not provided. Providing reliable and transparent sources (reconsidering their confidentiality) would offer a much more complete overview for further studies.

### Reporting the plastic fraction in separately collected waste

For certain waste categories, such as textiles and clothing and e-waste, the uncertainty lies in determining what happens to solely the plastic content of these products. Therefore, valuable data on the collection and processing of waste (e.g. Baldé et al., 2020; Hopstaken et al., 2020, and Stichting OPEN, personal communication, 2021) can be even more insightful with reports of waste management of plastic fractions only. This can only occur once centralisation of reporting occurs. For example, Stichting OPEN collects 50% of Dutch e-waste, but it is then sent to over 20 recycling facilities (Stichting OPEN, personal communication, 2021) and no one is responsible for assembling all the data.

### Reporting of recycled and non-recycled amounts

RWS has a large database of Dutch plastic waste collection and proper waste management of separately collected waste. To extend future MFA analyses beyond our ‘destinations’, one would need reports on which of the items sent to be recycled were in fact recycled. As of 1 July 2021, the Netherlands enforced the European Commission Decree 2019/665 (Overheid: Ministerie van Infrastructuur en Waterstaat, 2021) stating that for plastic packaging waste sent to recycling plants, the weight of residual moisture, dirt and non-packaging must be reported. Extending this regulation for all separately collected waste streams (including mixed waste) would provide a more accurate picture of total plastic recycling efficiency. Furthermore, determining which items (made of mixed plastics) or which types of virgin plastics should no longer be produced since they are not actually recycled would be valuable, especially those currently labelled as recyclable. Having reports on this non-recyclable content in waste streams can be used as leverage to change policies, for example to tax virgin fossil-based plastic and reduce taxes on recycled plastics (Friant et al., 2022).

### Litter monitoring programme recommendations

We suggest that all reporters of littered waste identify which items are plastic and weigh those items. Furthermore, it is highly recommended that fluxes of littered plastics ending up in the environment are measured, by collecting (and removing) litter over a given area and timespan, as in van Emmerik and de Lange (2021). This will allow for extrapolation of the data spatially (to a national scale) and temporally (to an annual scale). For example, RWS has a land litter monitoring project (Zwerfafval Rijkswaterstaat, 2022) that has been running for numerous years; however, the waste was not collected after the monitoring round and plastic items were not identified (RWS, personal communication, 2022) so we could not include their data. According to Eco Consult (project managers of the RWS project), from 2022 they do plan to identify and weigh plastic items.

Furthermore, addressing the exact origin of littered items is very complex. Though in this study our scope defines any items that are found on Dutch riverbanks or beaches as ‘Dutch litter’, there are many ways that foreign litter can end up on Dutch territory: (1) littered abroad and transported to the Netherlands by rivers or ocean currents, (2) foreigners come to the Netherlands and litter, and (3) Dutch residents go abroad and bring back foreign products and litter. A recent study suggests that the travel path length for riverine plastics is quite short and limited (Newbould et al., 2021); even after extreme discharge, there seems to be limited downstream transport. Further work could include designing a model to estimate pathways of foreign trash ending up as litter in the Netherlands.

## Conclusion

We present estimates for the known mass flow of Dutch plastic waste in 2017, including sources and destinations for properly managed and mismanaged waste. We use two models, one based on a material flow analysis using reported collection of plastic waste (mostly sent to be properly processed) and the other based on estimated mass of plastic waste on Dutch riverbanks and beaches, and inadequate management of exported waste in importing countries. We show that 1.9–2.1 million metric tonnes of Dutch plastic waste was known to be generated in 2017 and an equivalent of up to 0.6% of which entered the environment (within our scope). We highlight that the Netherlands has large import and export amounts of plastic scraps and there are insufficient centralised reports to trace what happens to them. We provide guidelines and recommendations that, though are focused on the Dutch waste system and policies, can also be applicable to other high-income countries to improve understanding of the unknowns of plastic waste generated, processed and mismanaged. Our main suggestions are that (1) waste management of traded plastic scraps should be tracked globally, (2) collection and the destination of the plastic fraction for all waste streams should be reported and (3) litter monitoring projects should provide data in terms of fraction of plastics, mass and flux. Implementing these suggestions will require efforts from policymakers, waste processing facilities and database managers on both national and international levels. Our recommendations can provide insights to stakeholders with goals to reach plastic circularity and a zero plastic pollution footprint within their own countries and, maybe more importantly, abroad.

## Supplemental Material

sj-pdf-1-wmr-10.1177_0734242X231180863 – Supplemental material for Knowns and unknowns of plastic waste flows in the Netherlands Click here for additional data file. Supplemental material, sj-pdf-1-wmr-10.1177_0734242X231180863 for Knowns and unknowns of plastic waste flows in the Netherlands by Delphine Lobelle, Li Shen, Bas van Huet, Tim van Emmerik, Mikael Kaandorp, Giulia Iattoni, Cornelius Peter Baldé, Kara Lavender Law and Erik van Sebille in Waste Management & Research

sj-pdf-2-wmr-10.1177_0734242X231180863 – Supplemental material for Knowns and unknowns of plastic waste flows in the Netherlands Click here for additional data file. Supplemental material, sj-pdf-2-wmr-10.1177_0734242X231180863 for Knowns and unknowns of plastic waste flows in the Netherlands by Delphine Lobelle, Li Shen, Bas van Huet, Tim van Emmerik, Mikael Kaandorp, Giulia Iattoni, Cornelius Peter Baldé, Kara Lavender Law and Erik van Sebille in Waste Management & Research
