# Supplementary material for: Knowns and unknowns of plastic waste flows in the Netherlands
Source: Waste Manag Res. 2023 Jul 16;42(1):27–40. doi: 10.1177/0734242X231180863 (PMC10759246; doi:10.1177/0734242X231180863)
Supplement: sj-pdf-1-wmr-10.1177_0734242X231180863 – Supplemental material for Knowns and unknowns of plastic waste flows in the Netherlands [file sj-pdf-1-wmr-10.1177_0734242X231180863.pdf]

Supplementary Table 1 - An overview of the results from Method 1 (Material Flow Analysis) and Method 2 (mismanagement model) showing the sources (in yellow) and destinations (in blue) of Dutch plastic waste, properly managed (in blue) and mismanaged (in orange), in 2017. Tables are separated into (a) average (b), low, and (c) high estimates, which are summarised in Table 3 in the main text using the average value, +/- the high and low estimates. The mismanagement model results are also shown in (d) for average, (e) for low, and (f) for high mismanagement estimates. Further breakdowns or details to reach all estimates are found in Tables S2-S6. All values in italics (the last two rows in Tables a-c) are not included to compute the total waste generated; note that summing the % Destination totals will not sum to 100% due to this (see Fig. 2 in the main text to see the intermediary steps of some waste streams being sent to recycling and then to landfills, for example). "NA" stands for not applicable, data sources are in column Y and other specific notes are in column Z.

(a) Average estimates

Source category #	Category	*Source uncertainty (not applicable for average estimates)*	% of total waste generated	Waste generated [ktons]	*Destination uncertainty (not applicable for average estimates)*	% sent to recycling	Amount sent to recycling [ktons]	% sent for energy recovery	Amount sent for incineration (energy recovery) [ktons]	% sent to landfill	Amount sent to landfill [ktons]	% sent to be reused	Amount sent to be reused [ktons]	% sent for export	Amount sent for export (unknown management) [ktons]	% sent to foreign waste facility	Amount sent to foreign waste facility [ktons]	% unreported	Unreported [ktons]	Littered on NL beaches [ktons]	Littered on NL river banks [ktons]	Inadequate management in importing country [ktons]	Data source	*Notes
M13	Imported plastic scraps	*NA*	31	**623**	*NA*	16%	100	0%	0	0%	0	0%	0	0%	0	0%	0	84%	523	NA	NA	0.0	UN Comtrade	
M1	Household plastic packaging	*NA*	20	**398**	*NA*	43%	169	57%	229	0%	0	0%	0	0%	0	0%	0	0%	0	NA	0.023	0.0	RWS + Brouwer et al. 2019	
M12	Textiles & clothing	*NA*	13	**250**	*NA*	1%	2	48%	121	0%	0	3%	7	2%	6	44%	109	2%	5	NA	0.002	3.0	RWS Ffact report	
M2	Household plastic non-packaging	*NA*	10	**203**	*NA*	14%	28	72%	146	2%	4	0%	0	0%	0	0%	0	12%	25	NA	0.005	0.0	RWS + Brouwer et al. 2019	
M4	Industry & manufacturing	*NA*	8	**157**	*NA*	90%	141	9%	14	1%	2	0%	0	0%	0	0%	0	0%	0	NA	0.000	0.0	RWS	
M6	Transport & storage	*NA*	5	**103**	*NA*	63%	64	5%	5	0%	0	32%	33	0%	0	0%	0	0%	0	NA	NA	0.0	RWS	
M5	Services & administration	*NA*	5	**91**	*NA*	70%	64	15%	13	15%	14	0%	0	0%	0	0%	0	0%	0	NA	2E-05	0.0	RWS	
M11	E-waste	*NA*	4	**75**	*NA*	38%	29	20%	15	1%	0	0%	0	3%	2	8%	6	30%	23	NA	NA	1.0	UNU/UNITAR	
M10	End-of-life vehicles	*NA*	2	**36**	*NA*	13%	5	66%	24	0%	0	0%	0	21%	7	0%	0	0%	0	NA	0.000	3.0	ARN	
M7	Agriculture, forestry & fishing	*NA*	2	**30**	*NA*	78%	23	0%	0	22%	7	0%	0	0%	0	0%	0	0%	0	NA	0.004	0.0	RWS	
M3	Building & construction	*NA*	1	**21**	*NA*	90%	19	0%	0	10%	2	0%	0	0%	0	0%	0	0%	0	NA	0.003	0.0	RWS	
M8	Water treatment & supply	*NA*	0.08	**2**	*NA*	100%	2	0%	0	0%	0	0%	0	0%	0	0%	0	0%	0	NA	NA	0.0	RWS	
M9	Energy & Mining	*NA*	0.06	**1**	*NA*	92%	1	8%	0	0%	0	0%	0	0%	0	0%	0	0%	0	NA	NA	0.0	RWS	
Loss	Littered (unreported source)	*NA*	0.01	**0.10**	*NA*	0%	0	0%	0	0%	0	0%	0	0%	0	0%	0	0%	0	0.02	0.08	0.0	Kaandorp et al. 2021, Van Emmerik et al. 2021	
	*Unreported**	*NA*	NA	*305*	*NA*	*0%*	*0*	*0%*	*0*	*0%*	*0*	*0%*	*0*	*100%*	*305*	*0%*	*0*	*0%*	*0*	*NA*	*NA*	*5.3*	UN Comtrade, expert opinion (ILT), this study	The source for the exported waste 383 kt is unknown/unreported, apart from coming from recycling facilities (78 kt below). The remaining 305 kt is not included in the total waste generated amount to avoid double-counting (it could come from other waste streams or imported waste.)
	*From recycling**	*NA*	NA	*117*	*NA*	*0%*	*0*	*0%*	*0*	*6%*	*39*	*0%*	*0*	*0%*	*0*	*12%*	*78*	*0%*	*0*	*0*	*NA*	*0.0*	RWS	Since this waste first passes through the Dutch recycling facilities, to avoid double-counting, these numbers are not added to the total waste generated
	**TOTAL**			**1990**		**33%**	**648**	**29%**	**567**	**3%**	**67**	**2%**	**40**	**16%**	**320.3**	**10%**	**193.5**	**29%**	**575.8**	**0.02**	**0.12**	**12.3**		
	Total generated in NL (without imports)			**1367**																				

(b) Low estimates (numbers for waste generated/source from the table above are decreased by the source uncertainty %, column D and destinations by the destination uncertainty, column G)

Source category #	Category	Source uncertainty (from Table 1 in text)	% of total waste generated	Waste generated [ktons]	Destination uncertainty (from Table 1 in text)	% sent to recycling	Amount sent to recycling [ktons]	% sent for energy recovery	Amount sent for incineration (energy recovery) [ktons]	% sent to landfill	Amount sent to landfill [ktons]	% sent to be reused	Amount sent to be reused [ktons]	% sent for export	Amount sent for export (unknown management) [ktons]	% sent to foreign waste facility	Amount sent to foreign waste facility [ktons]	% unreported	Unreported [ktons]	Littered on NL beaches [ktons]	Littered on NL river banks [ktons]	Inadequate management in importing country [ktons]	Data source	*Notes
M13	Imported plastic scraps	0%	33	**623**	100%	0%	0	0%	0	0%	0	0%	0	0%	0	0%	0	100%	623	NA	NA	0	UN Comtrade	
M1	Household plastic packaging	5%	20	**378**	10%	40%	152	54%	206	0%	0	0%	0	0%	0	0%	0	5%	20	NA	2E-03	0	RWS + Brouwer et al. 2019	
M12	Textiles & clothing	20%	11	**200**	50%	1%	1	30%	61	0%	0	2%	4	1%	3	27%	55	39%	77	NA	2E-04	1.5	RWS Ffact report	
M2	Household plastic non-packaging	5%	10	**193**	10%	13%	25	68%	131	2%	3	0%	0	0%	0	0%	0	17%	33	NA	5E-04	0	RWS + Brouwer et al. 2019	
M4	Industry & manufacturing	5%	8	**149**	10%	85%	127	9%	13	1%	2	0%	0	0%	0	0%	0	0%	0	NA	4E-05	0	RWS	
M6	Transport & storage	5%	5	**98**	10%	59%	58	5%	5	0%	0	30%	30	0%	0	0%	0	5%	5	NA	NA	0	RWS	
M5	Services & administration	5%	5	**87**	10%	67%	58	14%	12	14%	12	0%	0	0%	0	0%	0	0%	0	NA	2E-06	0	RWS	
M11	E-waste	10%	4	**67**	50%	21%	14	11%	8	0%	0	0%	0	1%	1	4%	3	61%	41	NA	NA	0.5	UNU/UNITAR	
M10	End-of-life vehicles	10%	2	**32**	10%	13%	4	66%	21	0%	0	0%	0	21%	7	0%	0	0%	0	NA	3E-05	1.5	ARN	
M7	Agriculture, forestry & fishing	5%	2	**28**	10%	74%	21	0%	0	21%	6	0%	0	0%	0	0%	0	6%	2	NA	4E-04	0	RWS	
M3	Building & construction	5%	1	**20**	10%	85%	17	0%	0	9%	2	0%	0	0%	0	0%	0	0%	0	NA	3E-04	0	RWS	
M8	Water treatment & supply	5%	0	**2**	10%	95%	1	0%	0	0%	0	0%	0	0%	0	0%	0	5%	0	NA	NA	0	RWS	
M9	Energy & Mining	5%	0	**1**	10%	100%	1	8%	0	0%	0	0%	0	0%	0	0%	0	-8%	0	NA	NA	0	RWS	
Loss	Littered (unreported source)	NA	0	**0.02**	0%	0%	0	0%	0	0%	0	0%	0	0%	0	0%	0	0%	0	0.01	0	0	Kaandorp et al. 2021, Van Emmerik et al. 2021	
	Unreported*	*NA*	NA	*305*	*NA*	*0%*	*0*	*0%*	*0*	*0%*	*0*	*0%*	*0*	*100%*	*305*	*0%*	*0*	*0%*	*0*	*NA*	NA	*0.7*	UN Comtrade, expert opinion (ILT), this study	The source for the exported waste 383 kt is unknown/unreported, apart from coming from recycling facilities (78 kt below). The remaining 305 kt is not included in the total waste generated amount to avoid double-counting (it could come from other waste streams or imported waste.)
	From recycling*	*NA*	NA	*117*	*NA*	*0%*	*0*	*0%*	*0*	*8%*	*39*	*0%*	*0*	*0%*	*0*	*16%*	*78*	*0%*	*0*	*0*	*NA*	*0.0*	RWS	Since this waste first passes through the Dutch recycling facilities, to avoid double-counting, these numbers are not added to the total waste generated
	**TOTAL**			**1878**		**26%**	**481**	**24%**	**456**	**3%**	**64**	**2%**	**33**	**17%**	**316**	**7%**	**136**	**43%**	**801**	**0.01**	**0.01**	**4.3**		
	Total generated in NL (without imports)			**1255**																				

(c) High estimates (numbers for waste generated/source from the table above are increased by the source uncertainty %, column D and destinations by the destination uncertainty, column G)

Source category #	Category	Source uncertainty (from Table 1 in text)	% of total waste generated	Waste generated [ktons]	Destination uncertainty (from Table 1 in text)	% sent to recycling	Amount sent to recycling [ktons]	% sent for energy recovery	Amount sent for incineration (energy recovery) [ktons]	% sent to landfill	Amount sent to landfill [ktons]	% sent to be reused	Amount sent to be reused [ktons]	% sent for export	Amount sent for export (unknown management) [ktons]	% sent to foreign waste facility	Amount sent to foreign waste facility [ktons]	% unreported	Unreported [ktons]	Littered on NL beaches [ktons]	Littered on NL river banks [ktons]	Inadequate management in importing country [ktons]	Data source	*Notes
M13	Imported plastic scraps	0%	30	**623**	100%	32%	200	0%	0	0%	0	0%	0	0%	0	0%	0	68%	423	NA	NA	0	UN Comtrade	
M1	Household plastic packaging	5%	20	**418**	10%	45%	186	60%	252	0%	0	0%	0	0%	0	0%	0	0%	0	NA	0.243	0	RWS + Brouwer et al. 2019	Since the destination uncertainty is larger than the source uncertainty, the "unreported" amount was negative here (-20: budget could not be closed) - we modified the unreported to 0
M12	Textiles & clothing	20%	14	**300**	50%	1%	3	61%	182	0%	0	3%	11	3%	9	55%	164	0%	0	NA	0.011	4.50	RWS Ffact report	Since the destination uncertainty is larger than the source uncertainty, the "unreported" amount was negative here (-68: budget could not be closed) - we modified the unreported to 0
M2	Household plastic non-packaging	5%	10	**213**	10%	15%	31	75%	160	2%	4	0%	0	0%	0	0%	0	8%	18	NA	0.068	0	RWS + Brouwer et al. 2019	
M4	Industry & manufacturing	5%	8	**165**	10%	94%	155	9%	16	1%	2	0%	0	0%	0	0%	0	0%	0	NA	0.002	0	RWS	
M6	Transport & storage	5%	5	**108**	10%	66%	71	5%	6	0%	0	34%	36	0%	0	0%	0	0%	0	NA	NA	0	RWS	Since the destination uncertainty is larger than the source uncertainty, the "unreported" amount was negative here (-5: budget could not be closed) - we modified the unreported to 0
M5	Services & administration	5%	5	**96**	10%	74%	71	15%	15	16%	15	0%	0	0%	0	0%	0	0%	0	NA	5E-05	0	RWS	
M11	E-waste	10%	4	**82**	50%	52%	43	27%	23	1%	1	0%	0	4%	3	11%	9	5%	4	NA	NA	1.5	UNU/UNITAR	
M10	End-of-life vehicles	10%	2	**39**	10%	13%	5	66%	26	0%	0	0%	0	19%	7	0%	0	2%	1	NA	0.001	4.6	ARN	
M7	Agriculture, forestry & fishing	5%	1	**31**	10%	82%	26	0%	0	23%	7	0%	0	0%	0	0%	0	0%	0	NA	0.024	0	RWS	Since the destination uncertainty is larger than the source uncertainty, the "unreported" amount was negative here (-1: budget could not be closed) - we modified the unreported to 0
M3	Building & construction	5%	1	**22**	10%	94%	21	0%	0	10%	2	0%	0	0%	0	0%	0	0%	0	NA	0.018	0	RWS	
M8	Water treatment & supply	5%	0	**2**	10%	105%	2	0%	0	0%	0	0%	0	0%	0	0%	0	0%	0	NA	NA	0	RWS	Since the destination uncertainty is larger than the source uncertainty, the "unreported" amount was negative here (-0.08: budget could not be closed) - we modified the unreported to 0
M9	Energy & Mining	5%	0	**1**	10%	96%	1	9%	0	0%	0	0%	0	0%	0	0%	0	0%	0	NA	NA	0	RWS	Since the destination uncertainty is larger than the source uncertainty, the "unreported" amount was negative here (-0.06budget could not be closed) - we modified the unreported to 0
Loss	Littered (unreported source)	NA	0	**0.50**	0%	0%	0	0%	0	0%	0	0%	0	0%	0	0%	0	0%	0	0.03	0.477	0	Kaandorp et al. 2021, Van Emmerik et al. 2021	
	Unreported*	*NA*	NA	*305*	*NA*	*0%*	*0*	*0%*	*0*	*0%*	*0*	*0%*	*0*	*100%*	*305*	*0%*	*0*	*0%*	*0*	*NA*	*NA*	*9.8*	UN Comtrade, expert opinion (ILT), this study	The source for the exported waste 383 kt is unknown/unreported, apart from coming from recycling facilities (78 kt below). The remaining 305 kt is not included in the total waste generated amount to avoid double-counting (it could come from other waste streams or imported waste.)
	From recycling*	*NA*	NA	*117*	*NA*	*0%*	*0*	*0%*	*0*	*5%*	*39*	*0%*	*0*	*0%*	*0*	*10%*	*78*	*0%*	*0*	*0*	*NA*	*0.0*	RWS	Since this waste first passes through the Dutch recycling facilities, to avoid double-counting, these numbers are not added to the total waste generated
	**TOTAL**			**2101**		**39%**	**815**	**32%**	**678**	**3%**	**70**	**2%**	**47**	**15%**	**324**	**12%**	**251**	**21%**	**445**	**0.03**	**0.84**	**20.4**		
	Total generated in NL (without imports)			**1478**																				

Mismanaged Dutch plastic waste estimates [tonnes]

(d) Average estimate mismanagement (plastic enterering the environment)

	L1	L2	L3		
**Category**	**NL beaches**	**NL river banks**	**Foreign env**	**TOTAL**	**% of total waste generated**
Mismanaged plastic waste	21	81	12311	**12413**	0.6
% of total mismanagement	0.2	0.7	99.2		

(e) Low estimate mismanagement (plastic enterering the environment)

	L1	L2	L3		
**Category**	**NL beaches**	**NL river banks**	**Foreign env**	**TOTAL**	**% of total waste generated**
Mismanaged plastic waste	14.0	8.1	4260.3	**4282**	0.2
% of total mismanagement	0.3	0.2	99.5		

(f) High estimate mismanagement (plastic enterering the environment)

	L1	L2	L3		
Category	**NL beaches**	**NL river banks**	**Foreign env**	**TOTAL**	**% of total waste generated**
Mismanaged plastic waste	27.0	843.1	20361.8	**21232**	1.0
% of total mismanagement	0.1	4.0	95.9		

Supplementary Table 2 - (a) Subcategories in household plastic packaging and non-packaging from the Rijkswaterstaat database (personal communication, RWS). The net mass waste generated in 2017 and destinations are shown. Examples of the items found in the categories are also shown in the final column. (b) A further breakdown of the types of items generated and classified within the municipal solid waste. Both gross and net weights are included where possible. Data from a report on mixed MSW (Rijkswaterstaat, 2021).

(a)

Category	Sub-category	Waste generated [net; kt]	% sent to recycling	Amount sent to recycling [kt]	% sent for energy recovery	Sent for incineration (energy recovery) [kt]	% sent to landfill	Sent to landfill [kt]	% unreported	Unreported [kt] **	Examples of items in this category
**Household plastic packaging**		**398.2**	**42.5**	**169.3**	**57.5**	**228.9**	**0.0**	**0.0**	**0.0**	**0.0**	
	Plastic in PMD (plastic, metal, drink cartons)	173.2	81.0	140.3	19.0	32.9	0.0	0.0	0.0	0.0	Plastic bottles, chocolate wrappers, crisp packets…
	Collected separately	17.1	81.0	13.9	19.0	3.2	0.0	0.0	0.0	0.0	Plastic bottles, chocolate wrappers, crisp packets…
	Within mixed Municipal Solid Waste (MSW)*	207.9	7.3	15.1	92.7	192.8	0.0	0.0	0.0	0.0	Plastic bottles, chocolate wrappers,(see Fig. S2b)
**Household plastic non-packaging**		**202.8**	**13.9**	**28.3**	**71.8**	**145.6**	**1.8**	**3.7**	**12.5**	**25.3**	
	Small waste (in mixed MSW)*	153.0	7.3	11.1	92.7	141.9	0.0	0.0	0.0	0.0	Toys, garbage bags (see Fig. S2b)
	Bulky waste (collected separately)	24.5	70.0	17.2	15.0	3.7	15.0	3.7	0.0	0.0	Garden furniture
	Bulky waste (in mixed MSW)	25.3	0.0	0.0	0.0	0.0	0.0	0.0	100.0	25.3	Garden furniture
Total		**601.0**	32.9	197.6	62.3	374.5	0.6	3.7	4.2	25.3	

(b)

Categories ***					% (gross) of total household MSW	Mass (gross) [kt]	% (net) of total household MSW	Mass (net) [kt]
Plastics in household mixed MSW					14.1	431	11.8	360.7
	Plastic packaging				9.1	278.2	6.8	**207.9**
		Bottles			2.3	69.39	1.5	44.6
			Bottles (bevarage)		0.97	29.65	0.6	16.8
				Bottles water/soft drinks	0.75	22.93		
				Bottles diary/juices	0.21	6.42		
			Bottles (non-beverage)		1.3	39.74	0.9	27.8
		Other plastic packaging			6.87	210.02	5.3	163.2
			Carrier bags		0.77	23.54		
			Sheets (all sorts)		2.4	73.37	2.6	79.5
			Foil laminate (aluminium)		0.45	13.76		
			EPS styrofoam		0.15	4.59	0.1	4.3
			Shape retaining containers		3.1	94.77	2.6	79.5
	Plastics non-packaging ^				5	152.9	5	**152.9**
		Non-packaging			2.6	79.5	2.6	79.5
		Garbage bags			2.4	73.4	2.4	73.4

Total household mixed MSW [kt] **3057**

References/notes

* 'Municipal solid waste' is defined as: Waste collected and treated by or for municipalities. It covers waste from households, including bulky waste, similar waste from commerce and trade, office buildings, public institutions and small businesses, as well as yard and garden waste, street sweepings, the contents of litter containers, and market cleansing waste if managed as household waste. The definition excludes waste from municipal sewage networks and treatment, as well as waste from construction and demolition activities (OECD, 2021)

** For the lower and upper estimates: 0-10% of total bulky waste in MSW (505 kilotons) is plastic (expert judgement; RWS). Most is wood and rubble. So 0 to 50 kilotons bulky plastic generated with an unreported destination.

*** Data extracted from Tables 3.4 and 4.4 in: Rijkswaterstaat (2021), 'Samenstelling van het huishoudelijk restafval, sorteeranalyses 2020:Gemiddelde driejaarlijkse samenstelling 2019'. URL: https://puc.overheid.nl/rijkswaterstaat/doc/PUC_633943_31/

^ Non-packaging is not cleaned so net mass cannot be estimated

Supplementary Table 3 - (a) The total amount of e-waste, fraction of plastic in e-waste and mass of plastic in e-waste [kt] within each EU-6 category of Waste generated from Electrical and Electronic Equipment (e-waste). The destinations for each category are provided. Examples of items found in each category are provided far right. (b) The waste generated and destination of the fraction of plastics in e-waste that are composed of Brominated Flame Retardants (BFRs) - they are all sent to hazardous waste incineration plants.

Plastics in e-waste

(a)

EU-6 Categories	Total e-waste [kt]	% of plastic in total e-waste	Plastic in e-waste generated * [kt]	% sent to recycling **	Amount sent to recycling [kt]	% sent for energy recovery ***	Amount sent for energy recovery (incineration) [kt]	% sent to landfill ***	Amount sent to landfill [kt]	% sent to be reused ***	Amount sent to be reused [kt]	% sent for export ***	Amount sent for export [kt]	% unreported	Unreported [kt]	Examples of items in this category^
I. Temperature exchange equipment	60.1	20.2%	12.11	41.0%	4.97	17.5%	2.12	0.50%	0.06	8.0%	0.97	2.7%	0.33	30.3%	3.67	Cooling and freezing equipment. Such as refrigerators, freezers, radiators, air conditioners, and heat pumps
II. Screens and monitors	38.9	17.2%	6.69	39.0%	2.61	19.0%	1.27	1.00%	0.07	8.0%	0.54	2.7%	0.18	30.3%	2.03	Televisions, monitors, laptops, notebooks, and tablets.
III. Lamps	2.9	14.4%	0.43	46.0%	0.2	11.0%	0.05	2.00%	0.01	8.0%	0.03	2.7%	0.01	30.3%	0.13	Fluorescent lamps, high intensity discharge lamps, and LED lamps.
IV. Large equipment	126.2	11.6%	14.66	40.0%	5.86	18.5%	2.71	0.50%	0.07	8.0%	1.17	2.7%	0.4	30.3%	4.44	Washing machines, clothes dryers, dishwashing machines, electric stoves, large printing machines, copying equipment, and photovoltaic panels.
V. Small equipment	107.2	28.0%	30.01	35.5%	10.65	23.0%	6.9	0.50%	0.15	8.0%	2.4	2.7%	0.81	30.3%	9.09	Vacuum cleaners, microwaves, ventilation equipment, toasters, electric kettles, electric shavers, scales, calculators, radio sets, video cameras, electrical and electronic toys, small electrical and electronic tools, small medical devices, small monitoring, and control instruments.
VI. Small IT	29.3	37.3%	10.9	40.5%	4.41	18.0%	1.96	0.50%	0.05	8.0%	0.87	2.7%	0.29	30.3%	3.3	Mobile phones, Global Positioning System (GPS) devices, pocket calculators, routers, personal computers, printers, and telephones.
**Total**	**364.6**	**20.5%**	**74.8**	**40.3%**	**28.7**	**17.8%**	**15.01**	**0.80%**	**0.41**	**8.0%**	**5.98**	**2.7%**	**2.02**	**30.3%**	**22.66**	

Fraction of plastics in e-waste that are composed of Brominated Flame Retardants

(b)

EU-6 Categories	Waste generated * [kt]	% sent to recycling **	Amount sent to recycling [kt]	% sent for energy recovery ***	Amount sent for energy recovery (incineration) [kt]	% sent to landfill ***	Amount sent to landfill [kt]	% sent to be reused ***	Amount sent to be reused [kt]	% sent for export ***	Amount sent for export [kt]	% unreported	Unreported [kt]
I. Temperature exchange equipment	0.12		0	100.0%	0.12		0		0		0		0
II. Screens and monitors	1.07		0	100.0%	1.07		0		0		0		0
III. Lamps	0		0	100.0%	0		0		0		0		0
IV. Large equipment	0.44		0	100.0%	0.44		0		0		0		0
V. Small equipment	2.7		0	100.0%	2.7		0		0		0		0
VI. Small IT	2.07		0	100.0%	2.07		0		0		0		0
**Total**	**6.4**			**100.0%**	**6.4**								

NACE: The Statistical classification of economic activities in the European Community,

References/notes

* *Forti et al. 2020, Huisman et al. 2017*

** *WeCycle (2021 annual report)*

*** *Personal communication, UNU & Stichting Open*

^ Directive 2012/19/EU of

Supplementary Table 4 - The sources and destinations of the synthetic component (plastic) in waste from textiles and clothing that are: (a) generated within the Netherlands and imported and (b) exported from the Netherlands. All data comes from the Ffact report: Hopstaken et al. 2020*. We assumed a 63% synthetic fraction based on the Fiber Year 2019 global data.**

Domestic (generated within the Netherlands) and imported waste

(a)

Category	Waste generated [ktons]	% sent to recycling	Amount sent to recycling [ktons]	% sent for energy recovery	Amount sent for energy recovery (incineration) [ktons]	% sent to be reused	Amount sent to be reused [ktons]	% sent to be exported	Amount sent to be exported [ktons]	% unreported	Unreported [ktons]
Separately collected	81.7	1.5%	1.2	10.2%	8.3	4.8%	3.9	80.3%	65.6	3.3%	2.7
Mixed with household MSW	106.5	0.0%	0	100.0%	106.5	0.0%	0	0.0%	0	0.0%	0
Imported*	61.9	1.5%	0.9	10.2%	6.3	4.8%	3	80.3%	49.8	3.3%	2
**Total**	**250.1**		**2.1**		**121.1**		**6.9**		**115.4**		**4.7**

**We assume the same treatment of imported textile waste as domestic waste*

Exported waste (including domestic and initially imported waste):

(b)

Category	Waste generated* [ktons]	% sent to recycling	Amount sent to recycling [ktons]	% sent for energy recovery	Amount sent for energy recovery (incineration) [ktons]	% sent to be reused	Amount sent to be reused [ktons]	% sent to be exported	Amount sent to be exported [ktons]	% unreported	Unreported [ktons]
Separately collected domestically, then exported	65.6	40.4%	26.5	0%	0	54.4%	35.7	-	-		3.4
Imported, then exported	49.8	40.4%	20.1	0%	0	54.4%	27.1	-	-		2.6
**Total**	**115.4***		**46.6**		**0**		**62.8**		**0**		**6**

** these "waste generated" values are the same as in J4 to J7*

**Synthetic fraction (plastic) in textiles and clothing assumed**:**	**63%**	*The Fiber Year (2019)*

References

* Hopstaken, F., van der Schalk, A., van der Mesen, M. & Custers, F. (2020), 'Massabalans 838 textiel 2018'. Ffact report. https://www.ffact.nl/wp-content/uploads/2020/04/Rapport-massabalans-textiel-2018-20200327.pdf

** The Fiber Year (2019), World Survey on Textiles Nonwovens .

Supplementary Table 5 - (a) the low, high and average estimated mass flux of plastic litter onto riverbanks throughout the Netherlands per year [kton/yr]. Our results are based on data collected of average mass and standard deviation of an item in each of our categories*. The number of items per 100m stretch was collected by volunteers, based on locations with at least 3 samples over the 2-year campaign (2017-2019)**. (b) the conversion table from the OSPAR classification of litter to our 13 categories.

(a)

Source category #	Category (after conversion from Table 5b)	Mean mass/item [g]*	Standard deviation mass/item [g]*	# items/100m/yr**	Mass flux/total length of NL riverbanks/yr [tonnes]	Upper estimate of mass flux (mean + 1 std in column D) [tonnes]	Lower estimate of mean flux (1 order of magnitude lower than the total in G) [tonnes]
M1	Household packaging	7.6	72.7	324.4	22.8	242.7	2.28
M2	Household non-packaging	8.1	106.6	71.8	4.8	67.8	0.48
M3	Building & construction	65.1	382.7	4.3	2.6	17.7	0.26
M4	Industry & manufacturing	15.5	70.5	3.0	0.4	2.4	0.04
M5	Services & administration	1.8	2.9	1.1	0.0	0.0	0.00
M6 & M10	ELV & vehicle parts	79.3	159.9	0.4	0.3	0.8	0.03
M7	Agriculture, forestry & fishing	24.7	120.2	18.4	4.1	24.2	0.41
M12	Textiles & clothing	24.6	113.8	8.8	2.0	11.0	0.20
	Other/fragments	17.0	167.4	284.7	44.0	476.6	4.40
	**Total [tonnes]**				**80.9**	**843.1**	**8.1**
	Total [kt]				0.08	0.84	0.01

References

* van Emmerik, T. & de Lange, S. (2021), 'Pilot monitoring drijvend zwerfafval en macroplas tics in rivieren'. URL: https://research.wur.nl/en/publications/075b13d7-4b8c-443f-a875-97e80b2156a5

** Roebroek, C. T., Hut, R., Vriend, P., Winter, W. D., Boonstra, M. & Emmerik, T. H. V. (2021), 'Disentangling variability in riverbank macrolitter observations', Environmental Science and Technology 55, 4932–4942.

**Total length of all Dutch rivers [km]**	**907.7**	*Dutch rivers include all bodies of water wider than 30 m (https://www.rijkswaterstaat.nl/water/vaarwegenoverzicht)*

Conversion table from OSPAR IDs to the current study's categories

(b)

OSPAR ID	ID	Description	Plastic type	This study's categories
**1**	plastic_6_packringen	Six pack ring	PO soft	Household packaging
**2**	plastic_tassen	Bag	PO soft	Household packaging
**3**	plastic_kleine_plastic_tasjes	Small bag	PO soft	Household packaging
**4.1**	plastic_drankflessen_groterdan_halveliter	Bottle (>= 0.5 L)	PET	Household packaging
**4.2**	plastic_drankflessen_kleinerdan_halveliter	Bottle (< 0.5 L)	PET	Household packaging
**4.3**	plastic_wikkels_van_drankflessen	Bottle label	PO soft	Household packaging
**5**	plastic_verpakking_van_schoonmaakmiddelen	Cleaning product packaging	PO hard	Household packaging
**6**	plastic_voedselverpakkingen_frietbakjes_etc	Food packaging	PS	Household packaging
**7**	plastic_cosmeticaverpakkingen	Cosmetics packaging	PO hard	Household packaging
**9**	plastic_motorolieverpakking_groterdan50cm	Motor oil packaging (>= 50 cm)	PO hard	Household packaging
**10**	plastic_jerrycans	Jerrycan	PO hard	Industry/manufacturing
**13**	plastic_kratten	Crate	PO hard	Household non-packaging
**14**	plastic_auto_onderdelen	Car parts	PO hard	ELV & vehicle parts
**15**	plastic_doppen_en_deksels	Caps and lids	PS	Household packaging
**16**	plastic_aanstekers	Lighter	PO hard	Household non-packaging
**20**	plastic_speelgoed	Toy	PS	Household non-packaging
**21**	plastic_plastic_bekers_of_delen_daarvan	Cup	PS	Household packaging
**24**	plastic_netzakken	Net bag	PO soft	Household packaging
**25**	plastic_handschoenen_huishoudelijk	Cleaning glove	PO soft	Household non-packaging
**113**	plastic_handschoenen_professioneel	Glove	PO soft	Household non-packaging
**31**	plastic_touw_diameter_groterdan_1cm	Rope	PO soft	Agriculture, forestry & fishing
**32**	plastic_touw_diameter_kleinerdan_1cm	Rope	PO soft	Agriculture, forestry & fishing
**35**	plastic_sportvisspullen	Fish gear	PO soft	Agriculture, forestry & fishing
**36**	plastic_breekstaafjes	Glowstick	PO hard	Household non-packaging
**38**	plastic_emmers	Bucket	PO hard	Household non-packaging
**40**	plastic_industrieel_verpakkingsmateriaal	Industrial packaging	PO soft	Industry & manufacturing
**42**	plastic_helmen	Helmet	PO hard	Building & construction
**43**	plastic_geweerpatronen	Gun rounds	PO hard	Household non-packaging
**57**	plastic_schoenen	Shoe	PO hard	Textiles & clothing
**117.1**	plastic_plastic_stukjes_0_2_5cm_hard_plastic	Hard fragment (< 5 cm)	PO hard	Other/fragments
**46.1**	plastic_plastic_stukjes_2_5_50cm_hard_plastic	Hard fragment (>= 5 cm)	PO hard	Other/fragments
**117.2**	plastic_plastic_stukjes_0_2_5cm_zacht_plastic	Soft fragment (< 5 cm)	PO soft	Other/fragments
**46.2**	plastic_plastic_stukjes_2_5_50cm_zacht_plastic	Soft fragment (>= 5 cm)	PO soft	Other/fragments
**48**	plastic_overig_plastic	Other plastic	Other plast	Other/fragments
**1172**	plastic_piepschuim_0_2_5cm	Foam fragment (< 5 cm)	EPS	Household packaging
**462**	plastic_piepschuim_2_5_50cm	Foam fragment (>=5 cm)	EPS	Household packaging
**6.1**	plastic_piepschuim_voedselverpakkingen	Foam food packaging	EPS	Household packaging
**47.1**	plastic_plastic_folies_groterdan_50cm	Foil (>= 50 cm)	PO soft	Household packaging
**47.2**	plastic_hard_plastic_groterdan_50cm	Hard other (>= 50 cm)	PO hard	Other/fragments
**22.1**	plastic_rietjes	Straw	PS	Household non-packaging
**19**	plastic_snoep_snack_chipsverpakking	Food wrapping	Multilayer	Household packaging
**472**	plastic_piepschuim_groterdan_50cm	Foam (> 50 cm)	EPS	Household packaging
**212**	plastic_piepschuim_bekers	Foam cup	EPS	Household packaging
**22**	plastic_bestek	Cutlery	PS	Household non-packaging
**481**	plastic_biofilm_waterfiltertjes	Water filter	PO hard	Household non-packaging
**11**	plastic_kitspuiten	Caulking gun	PO hard	Building & construction
**39**	plastic_kunststof_band_tiewraps	Cable tie	PO hard	Building & construction
**19.1**	plastic_lolliestokjes	Stick	PO hard	Household non-packaging
**8**	plastic_motorolieverpakking_kleinerdan50cm	Motor oil packaging (< 50 cm)	PO hard	Household packaging
**2.1**	plastic_vuilniszakken	Garbage bag	PO soft	Household non-packaging
**17**	plastic_schrijfwaren	Pen	PO hard	Household non-packaging
**35.1**	plastic_visdraad	Fishing wire	PO soft	Agriculture, forestry & fishing
**43.1**	plastic_vuurwerk	Firework	PO hard	Household non-packaging
**22.1**	plastic_borden_new	Plate	PS	Household non-packaging
**22.2**	plastic_roerstaafjes_new	Mixing stick	PS	Household non-packaging
**38.1**	plastic_bloempotten_new	Plant pot	PO hard	Household non-packaging
**39.1**	plastic_plakband_new	Tape	PO soft	Household non-packaging
**49**	rubber_ballonnen	Balloon	No plastic	Household non-packaging
**52**	rubber_banden	Tire	No plastic	ELV & vehicle parts
**53**	rubber_overig_rubber	Other rubber	No plastic	Other/fragments
**54**	textiel_kleding	Clothing	No plastic	Textiles & clothing
**55**	textiel_vloerbedekking	Carpet	No plastic	Textiles & clothing
**44**	textiel_schoeisel	Shoeware	No plastic	Textiles & clothing
**59**	textiel_overig_textiel	Other textile	No plastic	Textiles & clothing
**60**	papier_tassen	Paper bag	No plastic	NA
**61**	papier_karton	Carton	No plastic	NA
**63**	papier_sigarettenverpakking	Cigarette pack	No plastic	NA
**64**	papier_sigarettenfilters	Cigarette filter	No plastic	Household non-packaging
**65**	papier_kartonnen_bekers	Carton cup	No plastic	NA
**66**	papier_kranten	Newspaper	No plastic	NA
**67**	papier_papier_overig	Other paper	No plastic	NA
**62.1**	papier_drankkarton	Drink carton	No plastic	NA
**67.1**	papier_ondefinieerbaar	Other paper	No plastic	NA
**68**	hout_kurk	Cork	No plastic	NA
**69**	hout_pellets	Pellet	No plastic	NA
**72**	hout_ijsstokjes	Stick	No plastic	NA
**73**	hout_kwasten	Paintbrush	No plastic	NA
**74**	hout_overig_hout_keinderdan_50cm	Other wood (< 50 cm)	No plastic	NA
**75**	hout_overig_hout_groterdan_50cm	Other wood (>= 50 cm)	No plastic	NA
**81**	metaal_aluminiumfolie	Aluminium foil	No plastic	Household packaging
**81.1**	metaal_capsules	Metal capsule	No plastic	NA
**78**	metaal_drankblikjes	Drink can	No plastic	NA
**79**	metaal_elektriciteitsdraad	Electrical wire	No plastic	NA
**83**	metaal_oud_ijzer	Iron part	No plastic	NA
**77**	metaal_kroonkurken	Metal bottle cap	No plastic	NA
**84**	metaal_oliedrum	Oil drum	No plastic	NA
**88**	metaal_omheinigsdraad_prikkeldraad	Barbed wire	No plastic	NA
**76**	metaal_spuitbussen	Spray can	No plastic	NA
**86**	metaal_verfblik	Paint can	No plastic	NA
**80**	metaal_vislood	Fish lead	No plastic	NA
**82**	metaal_voedselblikken	Food can	No plastic	NA
**120**	metaal_wegwerpbarbecues	Single use grill	No plastic	NA
**89**	metaal_overig_metaal_kleinerdan_50cm	Other metal (< 50 cm)	No plastic	NA
**90**	metaal_overig_metaal_groterdan_50cm	Other metal (>= 50 cm)	No plastic	NA
**91**	glas_flessen_pottten	Pot	No plastic	NA
**92**	glas_lampen_tl_lampen	Tube lamp	No plastic	NA
**93**	glas_overig_glas	Other glass	No plastic	NA
**7**	sanitair_cosmetica	Cosmetics	No plastic	NA
**98**	sanitair_plastic_wattenstaafjes	Cotton swab	PO hard	Household non-packaging
**982**	sanitair_kartonnen_wattenstaafjes	Carton cotton swab	No plastic	NA
**102.2**	sanitair_vochtige_doekjes	Wet tissue	No plastic	NA
**97**	sanitair_condooms	Condom	No plastic	Household non-packaging
**99**	sanitair_maandverband_en_verpakkingen_ervan	Sanitary towel	No plastic	Household non-packaging
**18**	sanitair_plastic_kam_borstel	Hair brush	PO hard	Household non-packaging
**100**	sanitair_tampons_en_tamponapplicators	Tampon (applicator)	No plastic	Household non-packaging
**102.3**	sanitair_tissues_wc_papier	Toilet paper	No plastic	NA
**101**	sanitair_toiletverfrissers	Toilet refresher	PO hard	Household packaging
**102**	sanitair_overig_sanitair	Other sanitary	No plastic	NA
**103**	medisch_verpakkingen	Medical packaging	Multilayer	Services & administration
**104**	medisch_spuiten	Syringe	No plastic	NA
**105**	medisch_overig_medisch	Other medical	No plastic	NA
**999**	granulaat_korrels	Nurdle	Other plast	Industry & manufacturing

Supplementary Table 6 - (a) The countries importing plastic scraps that were exported from the Netherlands in 2017. The calculations to reach the high and low inadequate management estimates of plastic scraps in importing countries is also shown. All italic words are defined in the glossary in the main text and below the table. The method and definitions are adapted from Law et al. 2020***. (b) shows the other three separately collected waste streams that can be at risk of foreign inadequate management after export (we use the same 25-75% estimate as in Law et al. 2020). (c) As in (a) but for 2020.

	Column B	Column C	Column D	Column E	Column F	Column G	Column H	Column I	
(a)	**Destination of Dutch plastic scraps exported in 2017***	**Mass [kilotonnes]***	**% of total Dutch *plastic scraps* exported**	***% inadequately managed solid waste**** (Orange is >20%)	**Low estimate of *discarded* plastic scraps** (3% of orange non-EU countries in column C, 0% for EU countries) **[kilotonnes]**	**Low estimate of *discarded* plastic scraps** (13% of orange non-EU countries in column C, 9% for EU countries) **[kilotonnes]**	**Lower estimate of plastic scraps entering the environment of importing country (via *inadequate management*)** (25% of column F) **[kilotonnes]**	**Upper estimate of plastic scraps *entering the environment of importing country (via inadequate management)*** (75% of column G) **[kilotonnes]**	
1	**Germany**	**74.91**	**19.54**	**2.00**					
2	**China**	**68.92**	**17.98**	**23.30**	**2.07**	**8.96**	**0.52**	**6.72**	
3	**United Kingdom**	**60.59**	**15.80**	**2.60**					
4	**Belgium**	**46.58**	**12.15**	**2.30**					
5	**Ireland**	**24.15**	**6.30**	**3.00**					
6	**China, Hong Kong SAR**	**18.85**	**4.92**	**0.00**					
7	**France**	**11.90**	**3.10**	**0.00**					
8	**Italy**	**11.20**	**2.92**	**11.00**					
9	**India**	**9.96**	**2.60**	**77.00**	**0.30**	**1.30**	**0.07**	**0.97**	
10	**Viet Nam**	**9.24**	**2.41**	**62.00**	**0.28**	**1.20**	**0.07**	**0.90**	
11	Denmark	5.60	1.46	0.00					
12	Croatia	5.49	1.43	2.20					
13	Turkey	5.07	1.32	45.00	0.15	0.66	0.04	0.49	
14	Poland	4.79	1.25	0.00					
15	Malaysia	2.95	0.77	17.90					
16	Spain	2.84	0.74	0.00					
17	Czechia	2.80	0.73	0.00					
18	Other Asia, nes	2.27	0.59	23.25	0.07	0.30	0.02	0.22	
19	Romania	2.27	0.59	30.50	0.00	0.20	0.00	0.15	
20	Sweden	2.08	0.54	0.00					
21	Pakistan	1.52	0.40	70.00	0.05	0.20	0.01	0.15	
22	Indonesia	1.39	0.36	58.50	0.04	0.18	0.01	0.14	
23	Lithuania	1.07	0.28	1.40					
24	Rep. of Korea	1.05	0.27	0.00					
25	Portugal	0.82	0.21	0.00					
26	Latvia	0.80	0.21	15.70					
27	Austria	0.59	0.15	2.20					
28	Switzerland	0.43	0.11	0.00					
29	Luxembourg	0.42	0.11	0.30					
30	Finland	0.37	0.10	0.00					
31	South Africa	0.34	0.09	0.00					
32	Thailand	0.29	0.08	60.30	0.01	0.04	0.00217	0.03	
33	USA	0.28	0.07	0.00					
34	Singapore	0.26	0.07	0.00					
35	Russian Federation	0.23	0.06	95.50	0.01	0.03	0.00176	0.02	
36	Bulgaria	0.22	0.06	18.20					
37	Slovakia	0.19	0.05	5.70					
38	Greece	0.10	0.03	1.00					
39	Hungary	0.09	0.02	0.10					
40	Norway	0.08	0.02	1.40					
41	Slovenia	0.07	0.02	6.20					
42	Cyprus	0.07	0.02	7.60					
43	Iraq	0.07	0.02	100.00	0.00	0.01	0.00053	0.01	
44	Canada	0.07	0.02	0.00					
45	Malta	0.05	0.01	3.40					
46	Morocco	0.05	0.01	54.00	0.00	0.01	0.00035	0.00	
47	Curaçao	0.02	0.01	98.00	0.00	0.00	0.00015	0.00	
48	Estonia	0.02	0.01	12.90					
49	New Zealand	0.02	0.005	0.00					
50	Chile	0.01	0.002	13.80					
51	Suriname	0.003	0.001	100.00	0.00	0.00	0.00002	0.00	
52	Argentina	0.001	0.0004	22.60	0.00	0.00	0.00001	0.00	
53	Kenya	0.001	0.0003	92.00	0.00	0.00	0.00001	0.00	
54	Israel	0.0004	0.0001	0.00					
55	Saudi Arabia	0.0004	0.0001	0.00					
56	Bahrain	0.000004	0.000001	0.00					
	**TOTAL**	**383.4**	**100.0**				**0.74** (low inadequate management)	**9.81** (high inadequate management)	**5.28** average

(b)	**Separately collected waste exported**	**Total amount sent for export with unknown destination [kilotonnes]**	**Amount non-recyclable (if known) [kilotonnes]**	**Low inadequate management (25% of total) [kilotonnes]**	**High inadequate management (75% of total) [kilotonnes]**	**Average inadequate management (50% of non-recyclable) [kilotonnes]**	**Notes**
	ELVs (M10)	7.3	6.1	1.5	4.6	3.0	In NL, 87% of plastic in vehicles is sent to incineration plants due to low quality (ARN, personal comm.)
	E-waste (M11)	2.0	2.0	0.5	1.5	1.0	
	Textiles and Clothing (M12)	6.0	6.0	1.5	4.5	3.0	
	**TOTAL**			**3.5**	**10.6**	**7.0**	
	Total mismanagement abroad			**4.3**	**20.4**	**12.3**	

(c)	**Destination of Dutch plastic scraps exported in 2020***	**Mass [kilotonnes]***	**% of total Dutch *plastic scraps* exported**	***% inadequately managed solid waste**** (Orange is >20%)	**Low estimate of *discarded* plastic scraps** (3% of orange non-EU countries in column C, 0% for EU countries) **[kilotonnes]**	**Low estimate of *discarded* plastic scraps** (13% of orange non-EU countries in column C, 9% for EU countries) **[kilotonnes]**	**Lower estimate of plastic scraps entering the environment of importing country (via *inadequate management*)** (25% of column F) **[kilotonnes]**	**Upper estimate of plastic scraps *entering the environment of importing country (via inadequate management)*** (75% of column G) **[kilotonnes]**	
1	**Germany**	**89.46**	**21.65**	**2.00**					
2	**Belgium**	**67.13**	**16.25**	**2.30**					
3	**Indonesia**	**58.10**	**14.06**	**58.50**	**1.74**	**7.55**	**0.44**	**5.67**	
4	**Turkey**	**49.50**	**11.98**	**45.00**	**1.48**	**6.43**	**0.37**	**4.83**	
5	**United Kingdom**	**39.13**	**9.47**	**2.60**					
6	**France**	**19.13**	**4.63**	**0.00**					
7	**Poland**	**12.19**	**2.95**	**0.00**					
8	**Malaysia**	**11.42**	**2.76**	**17.90**					
9	**Viet Nam**	**10.15**	**2.46**	**62.00**	**0.30**	**1.32**	**0.08**	**0.99**	
10	**Italy**	**8.34**	**2.02**	**11.00**					
11	**Czechia**	**6.83**	**1.65**	**0.00**					
12	China, Hong Kong SAR	6.47	1.56	0.00					
13	Ireland	5.62	1.36	3.00					
14	Spain	4.91	1.19	0.00					
15	Serbia	3.79	0.92	43.81	0.11	0.49	0.03	0.37	
16	Pakistan	3.41	0.83	70.00	0.10	0.44	0.03	0.33	
17	Denmark	2.11	0.51	0.00					
18	Canada	1.97	0.48	0.00					
19	Rep. of Korea	1.96	0.47	0.00					
20	India	1.92	0.47	77.00	0.06	0.25	0.01	0.19	
21	Sweden	1.64	0.40	0.00					
22	Romania	0.90	0.22	30.50	0.03	0.12	0.01	0.09	
23	Finland	0.88	0.21	0.00					
24	Bulgaria	0.82	0.20	18.20					
25	Hungary	0.56	0.14	0.10					
26	Slovakia	0.51	0.12	5.70					
27	Estonia	0.47	0.11	12.90					
28	Luxembourg	0.45	0.11	0.30					
29	Brazil	0.42	0.10	23.21	0.01	0.05	0.00	0.04	
30	Mexico	0.38	0.09	21.00	0.01	0.05	0.00	0.04	
31	Slovenia	0.37	0.09	6.20					
32	Austria	0.28	0.07	2.20					
33	USA	0.28	0.07	0.00					
34	Bangladesh	0.22	0.05	94.75	0.01	0.03	0.00	0.02	
35	Latvia	0.14	0.04	15.70					
36	Other Asia, nes	0.14	0.03	23.25	0.00	0.02	0.00	0.01	
37	Lithuania	0.13	0.03	1.40					
38	Switzerland	0.11	0.03	0.00					
39	Greece	0.11	0.03	1.00					
40	Georgia	0.11	0.03	0.00					
41	Colombia	0.10	0.02	4.00					
42	China	0.09	0.02	23.25	0.00	0.01	0.00	0.01	
43	Montenegro	0.08	0.02	94.58	0.00	0.01	0.00	0.01	
44	Portugal	0.06	0.02	0.00					
45	United Arab Emirates	0.06	0.01	62.00	0.00	0.01	0.00	0.01	
46	Norway	0.06	0.01	1.40					
47	Israel	0.05	0.01	0.00					
48	Malta	0.05	0.01	3.40					
49	Philippines	0.03	0.01	72.00	0.00	0.00	0.00	0.00	
50	Cyprus	0.03	0.01	7.60					
51	Morocco	0.02	0.01	54.00	0.00	0.00	0.00	0.00	
52	Ukraine	0.02	0.01	47.04	0.00	0.00	0.00	0.00	
53	Costa Rica	0.02	0.005	9.10					
54	Belarus	0.02	0.005	7.10					
55	Nigeria	0.02	0.004	80.00	0.00	0.00	0.00	0.00	
56	United Rep. of Tanzania	0.02	0.004	100.00	0.00	0.00	0.00	0.00	
57	Russian Federation	0.01	0.003	95.50	0.00	0.00	0.00	0.00	
58	Japan	0.01	0.003	13.30					
59	Croatia	0.01	0.002	2.20					
60	Argentina	0.01	0.002	22.60	0.00	0.00	0.00	0.00	
61	Chile	0.004	0.001	13.80					
62	Thailand	0.003	0.001	60.30	0.00	0.00	0.00	0.00	
63	Australia	0.002	0.001	0.00					
64	Saudi Arabia	0.002	0.0005	0.00					
65	South Africa	0.002	0.0004	0.00					
66	Mongolia	0.001	0.0003	100.00	0.00	0.00	0.00	0.00	
67	Bosnia Herzegovina	0.001	0.0001	67.33	0.00	0.00	0.00	0.00	
68	Senegal	0.00002	0.000005	94.90	0.00	0.00	0.00	0.00	
	**TOTAL**	**413.23**	**100.00**				**0.97** (low inadequate management)	**12.61** (high inadequate management)	**6.79** average

References

* Data from UN Comtrade database (comtrade.un.org/data/). Accessed on January 18, 2022.

** Data from Kaza, S., Yao, L., Bhada-Tata, P. & Woerden, F. V. (2018), What a waste 2.0: a globalsnapshot of solid waste management to 2050, World Bank Publications.

*** Law, K. L., Starr, N., Siegler, T. R., Jambeck, J. R., Mallos, N. J. & Leonard, G. H. (2020),'The united states' contribution of plastic waste to land and ocean', Science Advances6, 1–8.

Glossary (definitions as per Law et al. 2020):**

Plastic scraps	Traded waste, parings and scrap, of plastic (commodity code: 3915) reported by the UN Comtrade database*
Inadequately managed waste	Solid waste that is not collected and/or properly contained because of lack of waste management infrastructure (Waste reported in "open dump," "waterways," "unaccounted for," and "other" categories in Kaza et al. 2018)
Discarded	Exported Dutch plastic scraps likely to be non-recyclable and therefore rejected or discarded by processing facilities, only in importing countries with an inadequate waste management over 20%
Inadequately managed plastic scraps	Discarded plastic scraps that end up in the environment (we assume 25-75% of the discarded amount, following Law et al. 2020)

Supplementary Table 7: The official European standard classification of productive economic activities (NACE Rev. 2) we use to classify the Dutch plastic waste generated and managed in this study. Annex 1, Section 8 in Regulation (EC) no 2150/2002 of the European Parliament and Council is used (*European Parliament, 2010)*

This study's classification	NACE Item Number	NACE Section/Division	NACE Description
Household plastic packaging & non-packaging	19	-	Waste generated by households
Building & construction	16	Section F	Construction
Industry & manufacturing	3–12	Division 10–33	Manufacture of items such as: food/beverages, tobacco, textiles, leather, paper, chemicals, rubber, plastic, furniture etc. and repair/installation of machinery and equipment.
Services & administration	17	Section G and Sections I–U	service activities such as: repair of vehicles, accommodation, communication, real estate, public administration and defence, education, entertainment, activities of extraterritorial organisations and bodies etc
Transport & storage	17	Section H	Transportation and storage
Agriculture, forestry & fishing	1	A	Agriculture, forestry and fishing
Water treatment & supply	14	36	Water collection, treatment and supply
Energy & Mining	2 and 13	B and D	Mining and quarrying. Electricity, gas, steam and air conditioning supply
