# Supplementary material for: Knowns and unknowns of plastic waste flows in the Netherlands
Source: Waste Manag Res. 2023 Jul 16;42(1):27–40. doi: 10.1177/0734242X231180863 (PMC10759246; doi:10.1177/0734242X231180863)
Supplement: sj-pdf-2-wmr-10.1177_0734242X231180863 – Supplemental material for Knowns and unknowns of plastic waste flows in the Netherlands [file sj-pdf-2-wmr-10.1177_0734242X231180863.pdf]

Supplementary Information: Knowns and unknowns of plastic waste flows in the Netherlands

1 Household plastic packaging waste (C1)

We isolate household waste from total collected 'post-consumer plastic packaging waste' (or *huishoudelijk kunststofverpakkingsafval*) since only the waste from households is weighed and reported by RWS. The waste collection of household plastic packaging waste (HPPW) in 2017 in the Netherlands was divided into three subcategories: (1) kerbside collection of plastics, metals, and beverage cartons waste (In Dutch: *plastic flessen, metalen verpakkingen en drankkartons* or PMD), in separate containers, (2) separately collected waste (in only a few municipalities, where plastic waste is collected in wheelie bins or plastic bags outside each house or residence, or dropped off directly at recycling centres by residents) and (3) co-collection of plastic waste with mixed municipal solid waste (MSW). Note that there are no enforced Dutch laws or fines for citizens to properly separate their waste (as can be the case in other EU countries).

The RWS database reports that for separately collected HPPW (subcategory 2 above), 81% was sent for recycling and 19% for energy recovery by incineration. Since the fraction of HPPW in PMD (subcategory 1) sent for recycling and energy recovery was not recorded, we assume that it was the same as above (81% and 19%, respectively). RWS did record the gross weight of plastics in HPPW PMD that was recycled in 2017 (144 kt). To attain the net weight (without residual contamination), we use the report from the Learning Centre Kunstof Verpakkingsafval in which 422 samples of PMD waste (from 17 municipalities) were analysed (Eijsbouts et al. 2018). They estimated that only 2.6% of plastic packaging waste was contaminated, hence a net weight of 140 kt of HPPW in PMD was recycled. As mentioned above, assuming that 140 kt represents 81% of the total plastics in PMD, we can compute the total (173 kt) and assume that the rest is incinerated for energy recovery (19% or 33 kt). For more details, see Table S2a.

The last waste stream for HPPW is plastic in mixed MSW. Fortunately, there are a lot of data available regarding the breakdown of household mixed MSW in the Netherlands (with subcategories down to the mass of water bottles and plastic sheets, for example; see Table

S2b). A report was conducted to estimate gross and net fractions of mixed MSW HPPW (see Table 3.4 and 4.4 in Rijkswaterstaat (2021)). Of the total mixed MSW household waste, plastic packaging accounted for 9.1% of the gross weight and 6.8% of the net weight (after cleaning out the moisture and contamination), which amounts to 208 kt (Rijkswaterstaat 2021). Since it is unknown how much is recycled and incinerated, we use estimated fractions from the Brouwer et al. (2019) model; 7.3% sent for recycling (15 kt) and 92.7% (193 kt) sent for incineration. This latter waste stream is the largest, meaning that 48% of the total HPPW produced in 2017 was sent for incineration from mixed MSW. In all three waste streams of HPPW, none of the waste was reported to be landfilled.

It is worth noting that Brouwer et al. (2019) mention that different waste flow calculation methods can reach different estimates. For example, the recycling rate from *Afvalfonds verpakkingen* (2017) that 50% (258 kt) of the plastic packaging put on the market was sent to recycling in 2017 reduces to 38% once residual contamination is accounted for and non-targeted plastic components are removed during the recycling process, following the new implementation decision (EU 2019/665; *Overheid: Ministerie van Infrastructuur en Waterstaat* 2021). For this reason, we aim to use net amounts as much as possible in our analysis.

2 Household plastic non-packaging waste (C2)

Non-packaging waste from households can be grouped into three categories based on size and collection method: (1) small non-packaging plastics in mixed MSW (such as garbage bags, plastic mugs and toys), (2) bulky plastic items in separately collected waste (such as garden furniture dropped off at recycling collection centres), and (3) bulky plastic items in bulky mixed MSW (such as garden furniture left outside households). Bulky items that do not fit in garbage bags or regular kerbside containers are collected by a different vehicle, hence the latter categorisation we use here.

For bulky non-packaging plastic waste collected separately, the RWS database reported 24.5 kt in total. They estimate that the majority (70%) is sent for recycling (17 kt), where private companies convert them to new items (e.g., converting old garden furniture into plant pots), 15% is sent to be incinerated (4 kt) and the rest (almost 4 kt) is sent to be landfilled (personal communication, RWS). For the non-packaging household waste found in mixed MSW, RWS does not clean and weigh small items (such as garbage bags), so we only have the gross weights (Table S2b). Contamination could therefore result in overestimation of these numbers. From gross amounts, about 5% (153 kt) of all mixed MSW was non-packaging plastics in 2017, of which about half was small non-packaging items and half was garbage bags (Rijkswaterstaat 2021). The total weight of bulky household mixed MSW was 505 kt; it is estimated that the majority (60-70%) was rubble and wood, however the fraction that is plastics is unknown so we assume that it is between 0 and 10% (0 to 50 kt). Since these items are too large for shredding, which is required prior to incineration, it is unknown whether

or not they are properly processed and this subcategory is placed into our flow analysis as ‘unreported’.

3 Separately collected plastic waste (C3–C9)

There is an extensive database of Dutch plastic waste separately collected from different economic activities (reported by RWS). The Dutch extended producer responsibility (EPR) scheme is called the ‘Packaging Waste Fund’ (in Dutch, *Afvalfonds Verpakkingen*). Plastic packaging producers and importers are obliged to pay an amount per kilogram of plastic produced (or imported) to the Fund to finance the collection, sorting, and recycling of their packaging waste (*Afvalfonds verpakkingen* 2017). They also fund litter clean-ups via “Nederland Schoon” (also seen in other countries, such as Norway; *Norwegian Retailers’ Environment Fund* 2022). Such schemes improve management and reporting of plastic packaging waste flows from all economic sectors.

Separately collected plastic waste consists of waste from: building & construction (C3), industry & manufacturing (C4), services & administration (C5), transport & storage (C6), agriculture & fishing (C7), water treatment & supply (C8), and energy & mining (C9). These seven waste streams come directly from the RWS database (personal communication, RWS) without any data post-processing or assumptions required. It is important to note that though most categories seem to cover post-consumer plastic waste, C4 and C5 can include pre-consumer plastic waste, too. Generally, these waste streams show that a large fraction is sent to recycling since they are collected separately. Any items from these waste streams that are found in mixed MSW are not reported and therefore not included in our model, so our results can be seen as a lower estimate of the total amount of Dutch plastic waste generated in 2017.

4 End-of-life vehicles (C10)

In this category, ‘vehicles’ are defined as passenger cars and light commercial vehicles with a gross vehicle weight up to 3,500 kg (and an average of 1,035 kg; personal communication, Auto Recycling Nederland; ARN). A vehicle reaches its end-of-life once it has completed its useful service and should be dismantled, shredded or disposed of correctly. The Directive 2000/53/EC and the Commission Decision 2005/293/EC provide regulations on ELVs (Williams et al. 2020). During ELV dismantling, parts that can be reused for vehicles in service are separated and the rest is sent for recycling, incineration or landfilled. Across Europe in 2017, 5.7 million ELVs were reported, about 206 thousand (3.6%) of which were in the Netherlands (Eurostat 2021).

ARN process 85% of all end-of-life vehicles (ELVs) in the Netherlands (*Auto Recycling*

Nederland 2022). They have provided tailored information for our study (personal communication, ARN) since the national statistics such as Eurostat (2021) do not yet publish data on plastic content and recycling of ELVs. In 2017, the average fraction of a Dutch ELV's mass that was made of plastics was 11.6% (120 kg for an average vehicle of 1,035 kg) and 234,595 cars were registered as ELVs (personal communication, ARN). This translates to a total of about 28.2 kt of plastic ELV waste was generated and collected in 2017. For the processing of ELV plastics, ARN's latest estimates of Dutch recycling and incineration rates (*ARN Holding B.V.* 2016), were based on amounts from 2016 but did not change significantly in 2017 (personal communication, ARN). 17% of the ELV plastic waste was sent to be recycled (4.7 kt) and the rest, 83%, was sent to be incinerated for energy recovery (23.5 kt). Reasons for this low recycling rate include that plastics coming from automotive shredder residues can carry a lot of dirt, dust, and other pollutants, as well as being heterogeneous in composition. To be able to compete with (cheaper) virgin plastics, automotive plastic producers need good quality, constant sources of sufficient quantity homogeneous plastic, which is challenging using the current post-ELV recycling process (personal communication, ARN). Another reason is that the use of composite plastics in cars (e.g. with glass fibre) or a mixture of many types of plastics (over 20 types) renders some plastics non-recyclable (personal communication, ARN). As a note, the fraction of plastic in vehicles is set to increase due to the growing demand of lightweight, cheaper, fuel-efficient vehicles so stricter regulations on the reuse or recycling of these plastics is required (Nagel 2016).

A surprising issue worth noting in this waste category is vehicles of unknown whereabouts. The location of 3.77 million European registered vehicles was listed as unknown in 2017 (Williams et al. 2020). In the Netherlands only about one in three of registered newly bought vehicles end up at a recycling facility 18 years later, and for the rest it is unknown if and how they are scrapped. Many of these vehicles are exported as used vehicles or ELVs (the export of vehicles does not fall under ARN's responsibility). Some may argue that exporting used vehicles is a form of circular economy. However, since there is no responsibility to track if and how Dutch ELVs are used in foreign countries (many of which might not have the facilities to properly recycle or reuse parts) the plastics in ELVs can be lost to the environment, hence why we apply a 25-75% loss to the environment after export. The report by *Netherlands Human Environment and Transport Inspectorate* (2020) identified an annual average (2018-2019) of 80,000 exported vehicles with an age above 16 years old. Upon inspection during their desk study, they found that the vehicles exported to West African countries were similar to vehicles dismantled in the Netherlands (based on age, emission class, mileage and expired road-worthiness tests). Following their approach, we use the CBS database (https://opendata.cbs.nl/) which provides the age and number of vehicles exported per year and determine 60,723 exported ELVs in 2017 with an age of 16 years old and above. Using the information from ARN that 11.6% of a vehicle's mass is plastic, an estimate of 7.3 kt of plastics in ELVs was exported in 2017. Furthermore, assuming that 87% of plastics

in ELVs is sent to waste incineration plants in the Netherlands, we estimate that 6.1 kt of exported ELV plastics are non-recyclable (Table S6b). This results in 1.5 - 4.6 kt of ELV plastic leakage abroad (with the average being 3 kt).

5 E-waste (C11)

We estimate the amount of plastics in Dutch Waste generated from Electrical and Electronic Equipment (WEEE; referred to as e-waste hereafter) using internal datasets from SCYCLE, co-hosted by the United Nations University/United Nations Institute for Training and Research (UNU/UNITAR). Specifically, we use the 'EEE Put on the Market' and e-waste generated from the Global E-waste Monitor 2020 (Forti et al. 2020) as well as information on the EEE material composition obtained from the ProSUM project (Huisman et al. 2017). We use the EU-6 categories defined in the official *European Union* (2012) e-waste report; temperature exchange equipment, screens and monitors, lamps, large equipment, small equipment and small IT (see the glossary in Table S7 for examples of each category). The total amount of e-waste (364.6 kt) is composed of 20.5% plastics (74.8 kt). The largest amount of plastics is found in small equipment such as vacuum cleaners, microwaves, toasters etc., where the plastic fraction amounts to 30 kt on average. Results show that for each category, the plastic fraction makes up between 11.6 and 37.3% (see Table S3a for the plastic fraction within each category).

Though the uncertainty of the data for the mass of plastics in e-waste generated mentioned above is low (10%), there are unfortunately no data on the waste management of the plastic content in Dutch e-waste. Reasons for this include that the plastic fraction is highly variable in e-waste items, and because each recycling facility can retrieve different types of materials, complicating the reporting process. The estimates we have are based on expert judgement during interviews with Stichting OPEN (hence a 50% uncertainty level; Table 1 in the main text), who have extensive data on the total e-waste treated in the Netherlands. Further informamtion on the estimate of total e-waste generated and properly managed in 2018 can be found in Baldé et al. (2020). Stichting OPEN (WeCycle until 2021) reports that in 2018, 50% of Dutch e-waste was collected by Stichting OPEN (https://jaarsite.Stichting OPEN.nl/2020/), and that these values were most likely very similar in 2017 (personal communication, Stichting OPEN). On average, 80% of what they collected was sent to be recycled by their 20-40 recycling facility partners (so 40% of the total e-waste generated), 9% was sent to be incinerated for energy recovery and 1% to be landfilled (for example, when it contains asbestos; personal communication, Stichting OPEN).

For the remaining 50% of e-waste that is not collected by Stichting OPEN, from Baldé et al. (2020) and personal communication with Stichting OPEN, the estimate is that: (1) 27% of the total e-waste is mixed with metal scraps that end up in scrap yards and are mostly shredded and traded (to Turkey or China, for example), (2) 8% is refurbished and

exported for reuse, consisting of mostly high-value items such as fridges, microwaves, IT servers and laptops, (3) 9% ends up in mixed MSW and is incinerated with energy recovery and (4) the rest is unreported. Since there is no current way to verify whether these numbers are representative of the processing of the plastic fraction in e-waste, we place a 50% uncertainty bound around our destination estimates. Since the 27% in metal scraps is likely mostly metal (an estimated guess is that only 10% of the content is plastics; personal communication, Stichting OPEN), we reach a total average of 40% sent for recycling, 18% sent for incineration, 1% landfilled, 2.7% exported with metal scraps, 8% fit for reuse and the remainder (about 30%) is unreported. As with the ELVs, an estimated 25-75% leakage to the foreign environment is applied to these 2 kt exported with metal scraps, with an unknown destination (hence 0.5–1.5 kt; see Table S6b). It is expected that since there are no landfills for e-waste in the Netherlands nor illegal dumping, the waste we classify as 'unreported' most likely does not end up in the environment in the Netherlands.

We have also included the fraction of plastics in e-waste that are made of Brominated Flame Retardants (BFRs) using Haarman et al. (2020). Regarding BFRs, being a hazardous material they are definitely not recycled and are instead sent to a hazardous waste incineration plant (see Table S3b).

6 Textiles & clothing (C12)

An extensive analysis of Dutch textile waste (Maldini et al. 2017) estimated that 80% of all post-consumer textiles are clothing and footwear and 20% are home textiles and other materials. Estimates of the mass balance of discarded household textiles in 2018 (Hopstaken et al. 2020) are presented in the Ffact report (commissioned by RWS). The data are not available for 2017, since their previous report was for 2012. Though this could result in uncertainties (since the rest of our MFA method is based on 2017), Method 2 (the leakage model) is based on data between 2017 and 2021, so 2018 fits within the total period of our overall analysis. The Ffact report was based on a questionnaire answered by 89 collectors and sorters of textiles as well as 800 second-hand shops to accurately map textiles sent for reuse as well. The Ffact report also provides estimates of what happens to exported textile waste, which is the only waste stream category where such detailed estimates are available. We did have to make an assumption on the fraction of textile and clothing made of plastics. We used 63%, since it was reported by *The Fiber Year* (2019) to be the global average fraction of fibres made of synthetic polymers. Without a country-specific estimate on the average fraction of polyester in Dutch clothes and textiles, we include a 20% uncertainty to the source and a 50% uncertainty to the destination (to match the reasoning used for e-waste; Table 1 in the main text). We also exclude footwear from the Ffact report in our analysis since we could not find a credible source for the average fraction of plastics in footwear (plus it makes up less than 5% of the total mass of 'textile waste' in their report).

The total amount of clothing, textiles and shoes disposed of in the Netherlands in 2018 was estimated to be 305 kt (10-28 kg per inhabitant) and an additional 98 kt of fibres were imported (Hopstaken et al. 2020). Using the 63% mentioned above, the total amount of synthetic fraction of the textile waste generated in the Netherlands is estimated at 188 kt in 2018 (Table S4a), where 43% was separately collected (about 82 kt) and 57% was found in mixed MSW household waste (about 106 kt). Of this domestic waste, the latter (mixed MSW) is completely sent to be incinerated for energy recovery and out of the separately collected waste, most is sent to be exported (80%), while the rest is sent for energy recovery (10%), recycling (1.5%), reuse (5%) and the remainder is unreported (3%). Regarding the import of textile waste in Hopstaken et al. (2020), about 62 kt of synthetic textiles were imported in 2018, and we assume that this waste is treated as is the domestic separately collected waste described above.

The exported synthetic waste amounted to 115 kt, which was a combination of waste generated domestically and originally imported into the Netherlands (66 kt and 50 kt, respectively; see Table S4b). Of the waste that is exported, a much larger fraction is sent for recycling (40%; 47 kt) than is recycled in the Netherlands (1.5%; 2 kt), as is the case for reuse (54%; 63 kt versus 5%; 7 kt domestically). The remaining 5% of the synthetic exported textile ends up unreported or discarded, amounting to about 6 kt of residual streams from textile sorting process that cannot be reused or recycled. There is insufficient information publicly available about how these textile sorting residual streams are treated in the foreign countries. We therefore assume that these residual streams face the risks of leakage. We apply the leakage range between 25-75% leading to a potential environmental leakage abroad of 3 kt on average, ranging from 1.5 kt to 4.5 kt.

7 Imported plastic scraps (C13)

The UN Comtrade reports a large amount of Dutch trade of plastic scraps, showing the highest import (623 kt) among EU28 countries and fourth highest export (383 kt) in 2017. For comparison, Germany imported 517 kt and exported 1,218 kt, and Belgium imported 192 kt and exported 324 kt (*United Nations* 2022). The Netherlands is by far the country with the highest net import of plastic scraps; 260 kt higher than the next highest in 2017, with the gap gradually decreasing to 110 kt higher in 2019 (*European Environment Agency* 2021). In 2019, however, the Netherlands was still seen to be importing and exporting a similar quantity of plastic scraps compared to the previous four years (Williams & McAndrew 2021).

Globally, there are two types of plastic scraps that are traded: recyclable and non-recyclable. Since recyclable plastic scraps have value and are sold to foreign countries, they are tracked and recorded in the Netherlands by the CBS (CBS 2019), hence we assume a negligible uncertainty for the source (Table 1 in the main text). However, one important finding from our study is that there is no report, literature, nor did any interview reveal what

happens to imported plastic scraps in the Netherlands; it does not appear to be tracked so it is unknown how much is properly managed in the Netherlands or re-exported. Personal communication with the Human Environment and Transport Inspectorate (ILT: *Inspectie Leefomgeving en Transport*) and the Dutch Federation of the Rubber and Plastic Industry (NRK: *De Federatie Nederlandse Rubber– en Kunststofindustrie*) suggest that even if the waste recorded by the UN Comtrade is recyclable, the recycling plants in the Netherlands do not have the capacity to process 623 kt of plastic scraps (it is closer to 200 kt). Although the UN Comtrade has the option to extract data for 're-exported', this is not available for the Netherlands (hence it has not been provided by the Dutch reporters, CBS). We therefore assume (with high levels of uncertainty; 100%) that of the 623 kt of plastic scraps imported, an estimate of 0-200 kt of the imported waste is sent to be recycled in the Netherlands and 423-623 kt are unreported. The 'unreported' destination could include incineration (for energy recovery) and re-export out of the Netherlands.

8 Legal framework for MFA sources and sinks

Before moving onto the leakage estimates, we provide an overview of the regulations linked to the sources and destinations of the MFA categories. Most of the recent legislative attention has been focused on single-use plastics from household plastic packaging. The responsibility falling on citizens to separate their waste into different containers was apparently not efficient enough, in areas of high population density. Therefore, some urban municipalities (since early 2022) have stopped separate collection schemes for lightweight packaging waste and have switched over to the mechanical recovery of plastics from mixed MSW, which results in higher yields and lower costs (Gradus 2020). As of January 1, 2021, the National (W)EEE Register states that all exported EEE for reuse needs to be notified (ILT started recording some data since 2019; personal communication, ILT). This does not specify that the exported plastic content in e-waste needs to be reported, however. There are currently limited regulations on textile waste processing on a European level. According to Directive 2008/98/EC separate collection for textiles by January 1 2025 should be set up (Article 10(2) and (3)). Furthermore, by 31 December 2024, the EU Commission will aim to set re-use and recycling targets for textile waste (*European Council* 2018).

9 Riverbank estimates (L1)

As explained in (van Emmerik et al. 2020), through the citizen science driven *Schone Rivieren* (Clean River) project, volunteers collected 152,415 anthropogenic litter items larger than 5 mm from 212 locations on the riverbanks of the Dutch Rhine-Meuse delta (see the sampling locations in Fig. 2 in van Emmerik et al. 2020). The items were categorised using the 110

categories of the OSPAR (Oslo and Paris conventions) Commission beached litter protocol (Wenneker & Oosterbaan 2010). Nine parent litter categories are identified (plastic, rubber, textile, wood, metal, glass, sanitary, and medical items, as well as 'unlisted'; see Wenneker & Oosterbaan 2010). This method was adapted for rivers by a Dutch non-governmental organisation *Stichting de Noordzee* (North Sea Foundation) and we re-categorised the plastic items to fit the 13 categories we use in this study (see the re-categorisations in Table S5b). Each monitoring location was sampled twice a year maximum, once in spring and once in autumn (these seasons were chosen to avoid data gaps due to riverbank vegetation and disturbing birds' breeding sites, as well as to increase data collection likelihood after peak discharge); up to 4 samples per location in total (van Emmerik et al. 2020). The sampling was done on 100 m long stretches of riverbank, with a maximum width of 25 m. For the exact width, the distance between the waterline and the high waterline was chosen, and therefore varied per location. See van Emmerik et al. (2020) for a more detailed description of the sampling techniques and how the locations were chosen.

Since the van Emmerik et al. (2020) method did not include the mass of sampled litter items, we use a dataset of over 14,000 sampled, weighed, and categorized litter items at seven locations along the IJssel, Meuse, and Rhine rivers (van Emmerik & de Lange 2021). The samples were collected monthly throughout 2021. All items were also categorised using the River-OSPAR protocol and weighed in the Wageningen University Water & Sediment lab.

10 Leakage after export (L3)

As with imports, there is a lack of tracking of exported plastic scraps in foreign countries. We interviewed two of the largest recycling companies in the Netherlands, responsible for a large fraction of the exports of plastic scraps (de Paauw Recycling B.V. and Kras Recycling). The Dutch Central Bureau for Statistics (CBS), Bishop et al. (2020) and the two recycling companies suggest that exported goods have value and plastic scraps are of high quality and recyclable. Though Law et al. (2020) revealed a high fraction of non-recyclable material in US waste bales (following the ISRI Scrap Specifications Circular (*Institute of Scrap Recycling Industries Inc.* 2018) classification of bales material by grade), the Netherlands has much stricter quality checks before shipping waste. Any so-called green-listed shipments can only have a maximum of 2% non-plastic contamination (see Article 4, point 3 in *Overheid: ILT* 2015). Dutch customs and ILT carried out around 800 inspections of green-listed plastic scraps for export in 2020 and less than 1% did not comply with the 2% level of contamination and was sent back to the exporter (personal communication, ILT). It is still legal to export non-green-listed waste, however there is an obligation to send a notification to RWS and ILT. The latest data available of this 'contaminated' exported waste is from 2015; 80 kt and 2016; 78 kt, where 90% of this contaminated waste was sent to Germany for sorting and incineration or recycling and the remainder to Austria, France and Poland (personal communication,

RWS). Without much difference between the 2015 and 2016 values, we assume that 78 kt were sent for foreign proper waste management in 2017 too. Of the 383 kt of Dutch plastic scraps exported in 2017, therefore, about 20% was shipped as contaminated waste with notifications, and sent for proper management abroad. Of the remaining 305 kt of exported plastic scraps, contaminated material could be up to 2% (*Overheid* 2022).

To further support our method for the estimates of plastic scraps ending up in the foreign environment, we refer to a report prepared for the Australian Department of Agriculture, Water and the Environment (*UTS* 2020) which includes interviews with local recyclers in Vietnam. When ranking the 56 countries that imported Dutch plastic scraps from most to least imported mass from the UN Comtrade database, Vietnam ranked 10th (and is also one of the 16 countries with inadequate waste management above 20%). Their report highlights that the import of plastics for recycling presents challenges due to lack of capacity and funding for monitoring and regulating plastic recycling operations. An estimated 90% of recycling in Vietnam is through informal household scale recycling activities in 'craft villages' (which is an important source of income for an estimated one million informal recyclers). These craft villages are dedicated to plastic sorting, washing and processing using old and low-tech techniques that can cause low-value micro and macro plastic to enter the environment; though the recyclable imported plastic scraps are less likely to be directly dumped in the environment, smaller pieces might be missed during manual sorting. Around 60% of the informal recyclers' plastic scrap feedstock is imported, since these sources are often more desirable than local waste (as shown in the main text, bales from the Netherlands are pre-sorted and recyclable scraps). Importing plastic scraps can therefore indirectly cause plastic pollution by removing the incentive to collect and separate local waste, which is exacerbated by the lack of a nationally coordinated system for waste management. For the scraps reaching formal recycling treatment facilities, they are running over capacity amd use old, low quality equipment (with inefficient leachate treatment or splitting systems), which can also cause environmental pollution. Any landfilled scraps are also at risk of ending up in the environment since 70% of Vietnam's landfills are registered as unsanitary. It is estimated that in Vietnam, almost all imported plastic scraps are converted into granulate or pellets, with 90% of the pellets being sold to China and the rest used domestically, though traceability of the pellets and total yield loss from import to re-export or reuse are not reported (*UTS* 2020). Following this report, we acknowledge that exported Dutch plastic scraps are likely to be of high quality and of financial interest to Vietnamese recyclers, however, the inefficient recycling facilities, unsanitary landfills and largely informal recycling system leads us to assume that our estimates of 0.75–9.75% of total Dutch plastic scraps could end up in the environment in Vietnam are reasonable.

References

Afvalfonds verpakkingen (2017), *Verpakkingen in de Circulaire Economie. Recycling verpakkingen Nederland 2017* .
URL: *https://afvalfondsverpakkingen.nl/a/i/Verpakkingen-in-de-circulaire-economie.pdf*

ARN Holding B.V. (2016), *Duurzaamheidverslag* .
URL: *https://arn.nl/over-arn/duurzaamheidsverslag/*

Auto Recycling Nederland (2022).
URL: *https://arn.nl*

Baldé, C., van den Brink, S., Forti, V., Schalk, A. V. D. & Hopstaken, F. (2020), 'The dutch weee flows 2020: What happened between 2010 and 2018?'.
URL: *https://www.scycle.info/the-dutch-weee-flows-2020-what-happened-between-2010-and-2018/*

Bishop, G., Styles, D. & Lens, P. N. (2020), 'Recycling of european plastic is a pathway for plastic debris in the ocean', *Environment International* **142**, 105893.
URL: *https://doi.org/10.1016/j.envint.2020.105893*

Brouwer, M., Picuno, C., van Velzen, E. U. T., Kuchta, K., Meester, S. D. & Ragaert, K. (2019), 'The impact of collection portfolio expansion on key performance indicators of the dutch recycling system for post-consumer plastic packaging waste, a comparison between 2014 and 2017', *Waste Management* **100**, 112–121.
URL: *https://doi.org/10.1016/j.wasman.2019.09.012*

CBS (2019), 'Less recyclable plastic waste sent to china'.
URL: *https://www.cbs.nl/en-gb/news/2019/11/less-recyclable-plastic-waste-sent-to-china*

Eijsbouts, R., Teeuw, C., Daamen, T. & Nieuwhof, S. (2018), 'Samenstelling ingezameld kunststof/pmd verpakkingen - het effect van inzamelsystemen'.
URL: *https://www.vang-hha.nl/nieuws-achtergronden/2018/onderzoek-fase-2/@203281/samenstelling/*

European Council (2018), *Directive 2008/98/CE of the European Parliament and of the Council of 19 November 2008 on waste and repealing certain Directives. Official Journal of European Union* **L312**, 1–59.
URL: *http://eur-lex.europa.eu/LexUriServ/LexUriServ.do?uri=OJ:L:2008:312:0003:01:ES:HTML*

European Environment Agency (2021), *Linking cross-border shipments of waste in the EU with the circular economy* .
URL: *https://www.eea.europa.eu/publications/linking-cross-border-shipments-of/linking-cross-border-shipments-of*

European Union (2012), *Directive 2012/19/EU of the European Parliament and of the Council of 4 July 2012 on waste electrical and electronic equipment (WEEE). Official Journal of the European Union* pp. 38–71.

Eurostat (2021), 'Eurostat database'.
URL: *https://ec.europa.eu/eurostat/web/main/data/database*

Forti, V., Baldé, C. P., Kuehr, R. & Bel, G. (2020), 'The global e-waste monitor 2020: Quantities, flows and the circular economy potential'.
URL: *http://ewastemonitor.info/*

Gradus, R. (2020), 'Postcollection separation of plastic recycling and design-for-recycling as solutions to low cost-effectiveness and plastic debris', *Sustainability* **12**, 1–12.

Haarman, A., Magalini, F. & Courtois, J. (2020), 'Study on the impacts of brominated flame retardants on the recycling of weee plastics in europe'.

Hopstaken, F., van der Schalk, A., van der Mesen, M. & Custers, F. (2020), 'Massabalans textiel 2018'.

Huisman, J., Leroy, P., Tertre, F., Söderman, M. L., Chancerel, P., Cassard, D., Amund, N., Wäger, P., Kushnir, D., Rotter, V. S., Mählitz, P., Herreras, L., Emmerich, J., Hallberg, A., Habib, H., Wagner, M. & Downes, S. (2017), 'Prospecting secondary raw materials in the urban mine and mining wastes (prosum) - final report'.
URL: *http://prosumproject.eu/sites/default/files/DIGITAL$_F$inal$_R$eport.pdf*

Institute of Scrap Recycling Industries Inc. (2018), *ISRI Scrap Specifications Circular* .
URL: *https://www.isri.org/recycling-commodities-old/scrap-specifications-circular*

Law, K. L., Starr, N., Siegler, T. R., Jambeck, J. R., Mallos, N. J. & Leonard, G. H. (2020), 'The united states' contribution of plastic waste to land and ocean', *Science Advances* **6**, 1–8.

Maldini, I., Duncker, L. & Bregman, L. (2017), 'Measuring the dutch clothing mountain: Data for sustainability-oriented studies and actions in the apparel sector'.
URL: *https://www.researchgate.net/publication/319902973*

Nagel, P. (2016), 'Cbi product factsheet: Plastics for vehicles in the european union'.
URL: $https://www.cbi.eu/sites/default/files/market_information/researches/product-factsheet-europe-plastics-vehicles.pdf$

Netherlands Human Environment and Transport Inspectorate (2020), *Used vehicles exported to Africa* pp. 1–61.

Norwegian Retailers' Environment Fund (2022).
URL: *https://handelensmiljofond.no/en/*

Overheid (2022), *Beleidsregel bestuursrechtelijke handhaving verontreinigd papier-, kunststof- en metaalafval 2015* .
URL: *https://wetten.overheid.nl/BWBR0037272/2015-12-04Artikel4*

Overheid: ILT (2015), *Staatscourant van het Koninkrijk der Nederlanden* .
URL: *https://zoek.officielebekendmakingen.nl/stcrt-2015-43221.html*

Overheid: Ministerie van Infrastructuur en Waterstaat (2021), *Staatsblad van het Koninkrijk der Nederlanden* .

Rijkswaterstaat (2021), 'Samenstelling van het huishoudelijk restafval, sorteeranalyses 2020: Gemiddelde driejaarlijkse samenstelling 2019'.
URL: *https://puc.overheid.nl/rijkswaterstaat/doc/$PUC_6 339433_3 1$/*

The Fiber Year (2019), *The Fiber Year 2019; World Survey on Textiles Nonwovens* .

United Nations (2022).
URL: *comtrade.un.org/data/*

UTS (2020), *Environmentally responsible trade in waste plastics Part 3: Case Studies on Plastic Waste Management and Trade in Asia Pacific, prepared for the Department of Agriculture, Water and the Environment by UTS Institute for Sustainable Futures & Asia Pacific Waste Consultants* .
URL: *https://www.dcceew.gov.au/sites/default/files/documents/ert-waste-plastics-report-3.pdf*

van Emmerik, T. & de Lange, S. (2021), 'Pilot monitoring drijvend zwerfafval en macroplastics in rivieren'.
URL: *https://research.wur.nl/en/publications/075b13d7-4b8c-443f-a875-97e80b2156a5*

van Emmerik, T., Roebroek, C., Winter, W. D., Vriend, P., Boonstra, M. & Hougee, M. (2020), 'Riverbank macrolitter in the dutch rhine-meuse delta', *Environmental Research Letters* **15**.

Wenneker, B. & Oosterbaan, L. (2010), 'Guideline for monitoring marine litter on the beaches in the ospar maritime area. edition 1.0.'.

Williams, R., Keeling, W., Petsinaris, F., Baron, Y. & Mehlhart, G. (2020), 'Supporting the evaluation of the directive 2000/53/ec on end-of-life vehicles'.
URL: *https://ec.europa.eu/environment/pdf/waste/elv/ELVD Evaluation-Final report Aug2020-rev1.pdf*

Williams, R. & McAndrew, K. (2021), 'Expanding the knowledge base on intra-eu waste movements in a circular economy'.
